# Recent Advances in Deep Learning-Based Spatiotemporal Fusion Methods for Remote Sensing Images

**DOI:** 10.3390/s25041093

**Published:** 2025-02-12

**Authors:** Zilong Lian, Yulin Zhan, Wenhao Zhang, Zhangjie Wang, Wenbo Liu, Xuhan Huang

**Affiliations:** 1 Aerospace Information Research Institute, Chinese Academy of Sciences, Beijing 100094, China; kingsleylin@stumail.nciae.edu.cn (Z.L.); wangzhangjie12@163.com (Z.W.); huangxuhan24@mails.ucas.ac.cn (X.H.); 2 School of Remote Sensing and Information Engineering, North China Institute of Aerospace Engineering, Langfang 065000, China; zhangwh@radi.ac.cn (W.Z.); liu2728210141@163.com (W.L.); 3 Hebei Collaborative Innovation Center for Aerospace Remote Sensing Information Processing and Application, Langfang 065000, China

**Keywords:** multi-sensor data fusion, deep learning, remote sensing images, temporal resolution, spatial resolution

## Abstract

Remote sensing images captured by satellites play a critical role in Earth observation (EO). With the advancement of satellite technology, the number and variety of remote sensing satellites have increased, which provide abundant data for precise environmental monitoring and effective resource management. However, existing satellite imagery often faces a trade-off between spatial and temporal resolutions. It is challenging for a single satellite to simultaneously capture images with high spatial and temporal resolutions. Consequently, spatiotemporal fusion techniques, which integrate images from different sensors, have garnered significant attention. Over the past decade, research on spatiotemporal fusion has achieved remarkable progress. Nevertheless, traditional fusion methods often encounter difficulties when dealing with complicated fusion scenarios. With the development of computer science, deep learning models, such as convolutional neural networks (CNNs), generative adversarial networks (GANs), Transformers, and diffusion models, have recently been introduced into the field of spatiotemporal fusion, resulting in efficient and accurate algorithms. These algorithms exhibit various strengths and limitations, which require further analysis and comparison. Therefore, this paper reviews the literature on deep learning-based spatiotemporal fusion methods, analyzes and compares existing deep learning-based fusion algorithms, summarizes current challenges in this field, and proposes possible directions for future studies.

## 1. Introduction

With the development of remote sensing technology, remote sensing images captured by satellites have been widely used in fields such as agriculture [1,2,3], ecology [4,5,6], and Earth surface observation [7,8]. However, current satellite images still fall short of meeting the need for high-resolution observations in dense time series and large areas. Due to constraints in sensor technology and the cost of satellite launches, there is a trade-off between the temporal and spatial resolutions of remote sensing satellites, which makes it difficult for satellites to achieve both high spatial and temporal resolutions at the same time. Additionally, restricted by swath width and weather conditions, it is challenging for high-spatial-resolution satellites to capture seamless images in large areas. Therefore, an economical and effective approach is to perform spatiotemporal fusion (STF) for remote sensing images to obtain images with both high spatial and high temporal resolutions. Spatiotemporal fusion is a technique that integrates high-spatial-resolution but low-temporal-resolution images with high-temporal-resolution but low-spatial-resolution images to create synthetic images with both high spatial and high temporal resolutions.

**Figure 1:**
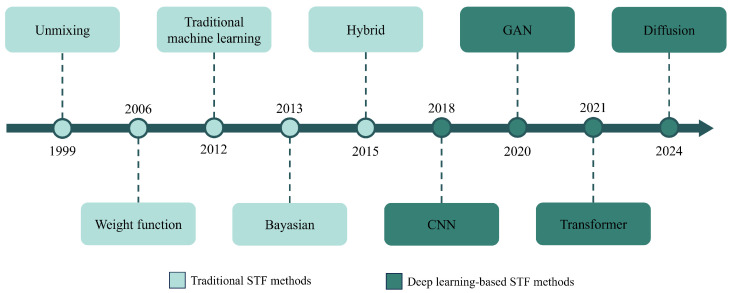
Development of spatiotemporal fusion methods.

Over the past decade, traditional spatiotemporal fusion research has made significant progress, and a series of traditional fusion methods have been developed (Figure 1). According to fusion mechanisms, traditional spatiotemporal fusion methods can be classified into unmixing-based, weight function-based, Bayesian-based, learning-based, and hybrid methods [9]. Each type of method is based on different principles. For example, unmixing-based methods perform spatiotemporal fusion using the linear spectral mixing theory [10,11]. Weight function-based methods establish a relationship between high-resolution and low-resolution pixels through weight functions [12,13,14]. Bayesian methods regard spatiotemporal fusion as a maximum a posteriori problem [15]. Learning-based methods construct spatial and temporal relationships using machine learning algorithms [16]. Hybrid methods combine multiple traditional methods to improve fusion accuracy [17]. Despite the distinct characteristics of these methods, they still face obstacles in practical applications. Traditional methods often rely on prior knowledge for model construction and require specific adjustments when applied to different regions. These limitations have hindered the further development and broader adoption of traditional methods.

**Figure 2:**
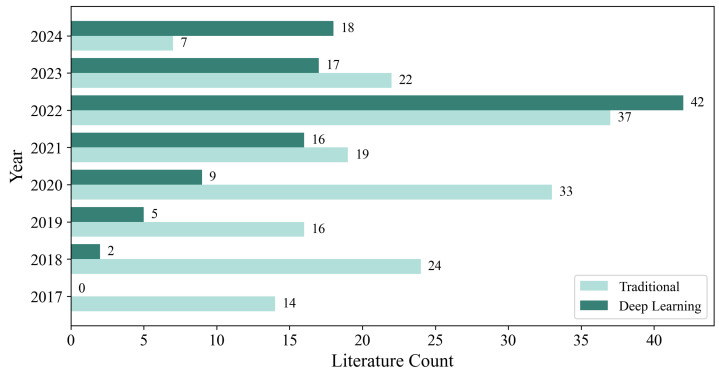
Yearly paper count of spatiotemporal fusion methods.

Therefore, to address the challenges associated with traditional fusion methods, deep learning techniques have been introduced into the field of spatiotemporal fusion. Compared to traditional methods, deep learning models excel in automated feature extraction, nonlinear modeling, and model generalization. These advantages endow deep learning models with significant potential for improving fusion performance. In the early stages, deep learning-based fusion algorithms mainly employed simple backpropagation networks [18,19,20] to improve traditional methods. By utilizing multi-layer network structures and nonlinear activation functions, these networks automatically extracted features and modeled nonlinear relationships between images. However, simple backpropagation networks could not further improve fusion results in complex scenarios. Consequently, more advanced models, such as convolutional neural networks (CNNs) [21], generative adversarial networks (GANs) [22], Transformers [23], and diffusion models [24], have gradually been applied in the field of spatiotemporal fusion. Compared to traditional methods, deep learning-based models demonstrate higher accuracy and efficiency in handling diverse fusion scenarios and have progressively become mainstream methods in spatiotemporal fusion (Figure 2).

Despite the significant advantages and potential of deep learning methods in spatiotemporal fusion, current review studies predominantly focus on traditional methods [9,25], while systematic reviews of deep learning approaches remain relatively scarce. This lack of comprehensive reviews has hindered the further development of deep learning-based spatiotemporal fusion methods. In response, this paper conducts a comprehensive survey of existing deep learning-based fusion methods, provides an in-depth analysis of various deep learning-based approaches, and thoroughly reviews the research progress and current status of these methods. Then, a quantitative analysis of the evaluation and application of deep learning-based fusion methods is carried out based on specific methods and examples. By categorizing and comparing different methods, this paper identifies the key challenges currently faced in the field of deep learning-based spatiotemporal fusion and offers perspectives on future research directions. These analyses and summaries aim to serve as references for subsequent research on spatiotemporal fusion, fostering further exploration and development of deep learning in this field.

## 2. Deep Learning-Based Spatiotemporal Fusion Methods

Before the advent of deep learning methods, traditional spatiotemporal fusion approaches faced problems such as complexity and difficulty in model design and application. For instance, constrained by linear spectral mixing theory, unmixing-based methods lack variability within coarse pixels and require prior classification, greatly limiting their applicability. Weight function-based methods rely heavily on prior knowledge in model design, resulting in reduced stability. Bayesian methods are computationally intensive, making them inefficient when capturing large-scale or high-resolution images. Learning-based methods rely heavily on complex hand-crafted features and, therefore, exhibit poor stability. Hybrid methods increase computational complexity and the difficulty of parameter tuning, often resulting in error propagation. These limitations have significantly constrained the performance and broader adoption of traditional spatiotemporal fusion methods.

**Figure 3:**
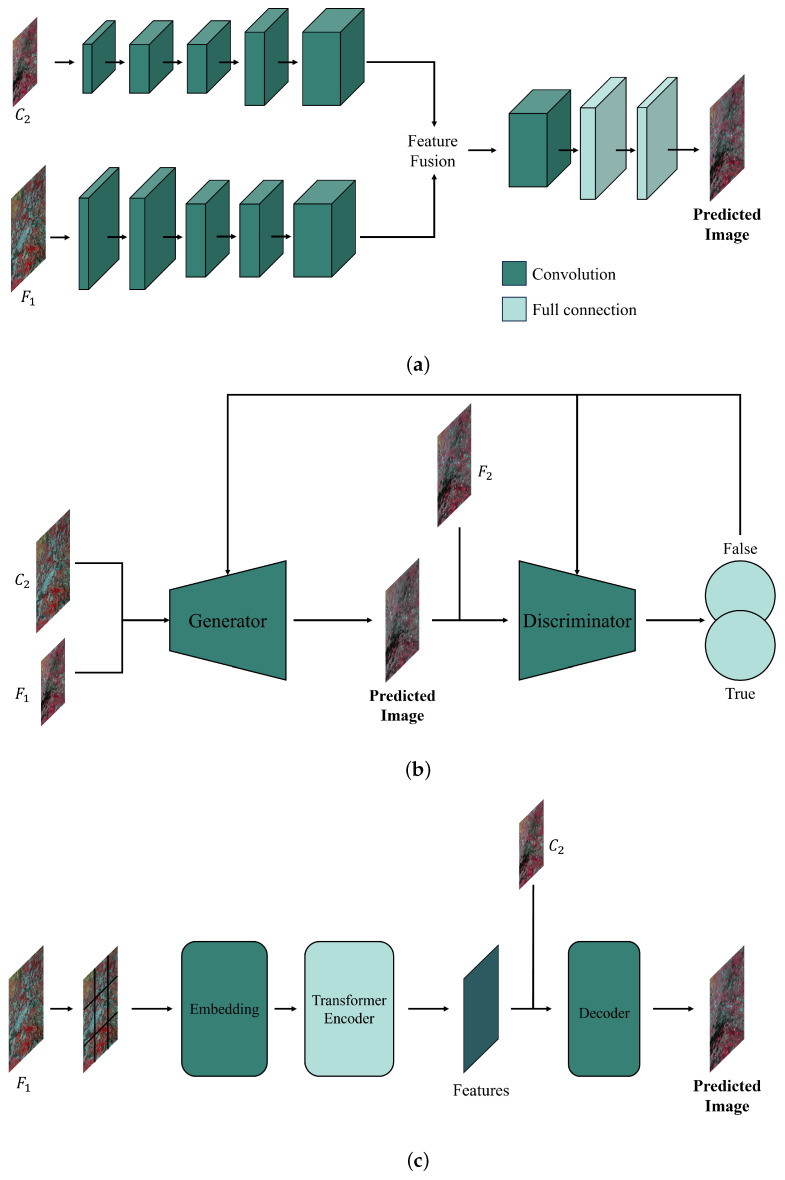

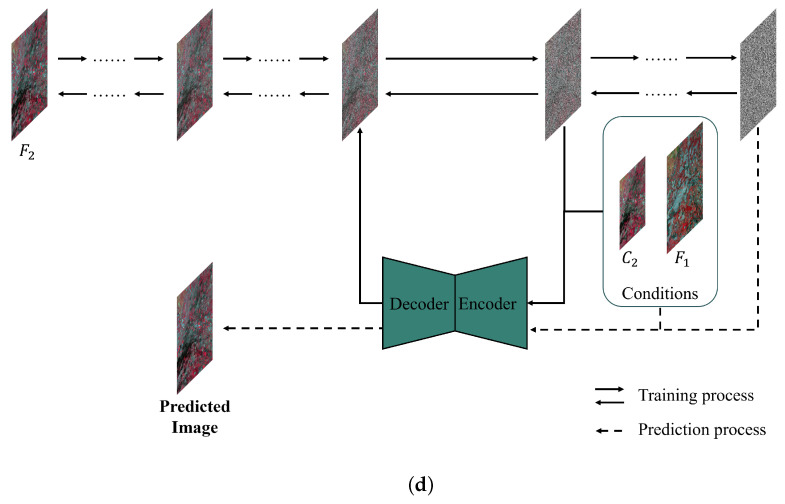
Example of deep learning-based spatiotemporal fusion (STF) models. (**a**) A CNN-based STF model (e.g., DCSTFN [26]). (**b**) A GAN-based STF model (e.g., GANSTFN [27]). (**c**) A Transformer-based STF model (e.g., MSNet [28]). (**d**) A diffusion-based STF model (e.g., STFDiff [29]). “F” and “C” represent fine images and coarse images, respectively. Subscripts “1” and “2” represent the reference date $t_{1}$ and the predicted date $t_{2}$, respectively.

To address the challenges in traditional spatiotemporal fusion, deep learning techniques have been introduced. Based on neural network architectures, these methods fall into four categories: convolutional neural network (CNN)-based, generative adversarial network (GAN)-based, Transformer-based, and diffusion-based methods. Each category leverages unique principles to enhance the accuracy and applicability of spatiotemporal fusion. CNNs improve fusion performance by handling image details and extracting features through local receptive fields and weight-sharing mechanisms. GANs generate high-quality images from limited data through adversarial learning between generators and discriminators, enhancing applicability. Transformers, with self-attention mechanisms, boost efficiency and accuracy in handling long temporal sequences and global spatial relationships. Diffusion models use diffusion and denoising processes to produce realistic, stable images. The adoption of deep learning techniques overcomes the limitations of traditional fusion methods and broadens their applicability in complex scenarios. This section categorizes and summarizes CNN-based, GAN-based, Transformer-based, and diffusion-based methods (Figure 3), providing an in-depth analysis of their strengths and weaknesses.

### 2.1. CNN-Based Fusion Methods

Originally proposed by [30], convolutional neural networks are designed for feature extraction. CNNs are a type of deep learning architecture that is particularly suitable for image-related tasks. Using convolutional layers, a CNN can extract local features from input images. The core advantage of a CNN is that it can automatically learn image features without manual feature extraction. In spatiotemporal fusion, CNNs effectively address the limitations of traditional methods in three areas: (1) Traditional methods rely on hand-crafted features, making it difficult to capture complex spatiotemporal characteristics, while CNN-based methods improve feature extraction efficiency and accuracy through automated learning. (2) Traditional methods use linear or simple nonlinear models that struggle to represent intricate spatial and temporal relationships, whereas CNNs, with multiple convolutional layers and nonlinear activation functions, provide strong nonlinear modeling capabilities. (3) Traditional methods suffer from low computational efficiency when processing large-scale data, whereas CNN-based methods excel in handling such data through parallel computation, thus enhancing spatiotemporal fusion performance.

Compared to traditional fusion methods, CNN-based methods offer clear advantages in feature extraction, fusion accuracy, and processing efficiency. Subsequent studies have further enhanced CNNs by incorporating residual connections and attention mechanisms, resulting in even greater performance. For example, residual blocks allow for deeper network layers to extract more complex features [31], and attention mechanisms reduce feature redundancy, improving computational efficiency [32]. Therefore, the CNN-based spatiotemporal fusion methods summarized in this paper are categorized into conventional CNN methods, residual-based CNN methods, and attention-based CNN methods, as shown in Table 1.

#### 2.1.1. Conventional CNN Methods

**Figure 4:**
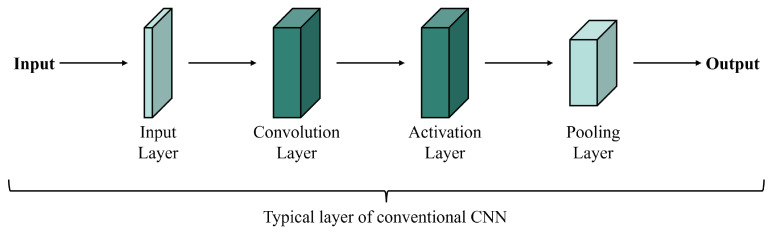
An example layer of a conventional CNN.

Conventional CNN methods use the basic CNN structure to perform fusion tasks. By processing each layer (Figure 4), these methods automatically capture local features from input images. The input layer receives the original image data as pixel values for each band. Convolutional layers then extract local features like edges, lines, and textures. Activation layers apply nonlinear functions to the convolution outputs, enabling the network to learn more complex features. Pooling layers reduce data dimensions and computational costs. Finally, fully connected layers integrate all features, and the output layer produces the predicted image.

Through these operations, conventional CNN-based fusion methods mitigate the complexity and instability associated with hand-crafted features in traditional methods. For example, STFDCNN [33] introduces a fusion model combining a nonlinear mapping CNN (NLMCNN) and a super-resolution CNN (SRCNN). NLMCNN trains feature extraction filters, nonlinear mapping filters, and reconstruction filters to extract features from coarse images, map them into residual feature maps, and reconstruct downsampled fine images. SRCNN correlates the output image with the original fine image. During training, NLMCNN learns the relationship between coarse and downsampled fine images, while SRCNN learns the relationship between the downsampled and original fine images. During prediction, STFDCNN uses the transition image from NLMCNN as input to the trained SRCNN to generate the predicted fine image. By separating spatial and temporal relationships into separate learning processes within two convolutional networks, STFDCNN significantly enhances fusion accuracy.

**Figure 5:**
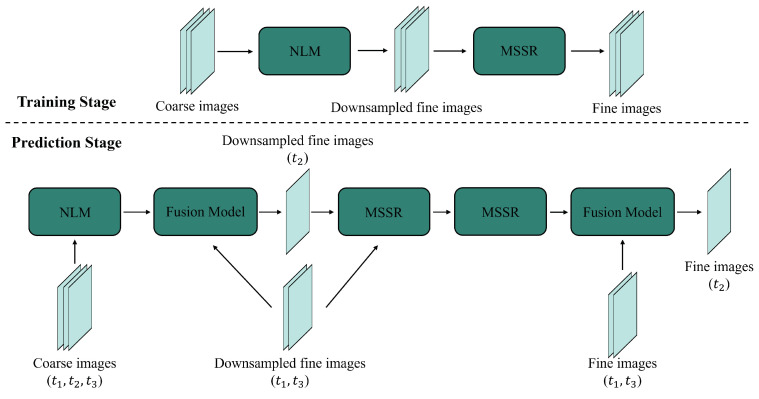
Flowchart of VDCN [35].

However, as the first conventional CNN-based spatiotemporal fusion model of its kind, STFDCNN has limitations. Subsequent studies focused on addressing these flaws. Zheng et al. [35] noted that STFDCNN’s three hidden layers struggle to capture complex nonlinear relationships and proposed the VDCN model. VDCN trains a deep NLM CNN between coarse images and downsampled fine images, followed by a deep multi-scale super-resolution (MSSR) CNN that correlates downsampled and original fine images. During prediction (Figure 5), MSSR CNN divides the downsampling into two stages to mitigate resolution gap issues. Also based on SRCNN, ESRCNN [37] resamples images to a uniform resolution before fusion. STFDCNN, focusing on nonlinear mappings, neglects temporal change information. To address this, DL-SDFM [41] generates feature maps that capture both temporal changes and spatial information. Using a two-stream convolutional network, DL-SDFM predicts phenological and land-cover changes separately. To improve robustness against phenological changes, LSTM-SRCNN [82] integrates long short-term memory (LSTM) networks with CNNs, assessing model performance across different phenological scenarios. Similarly, TSSTFN [83] employs LSTM to capture long-term dependencies. These conventional CNN-based methods progressively resolve issues related to nonlinear mapping, spatial detail reconstruction, and phenological changes, significantly enhancing spatiotemporal fusion accuracy and robustness.

Despite the improvements made by the above methods, conventional CNN-based approaches still have areas needing enhancement. Subsequent research has further improved fusion performance. For instance, feature-level fusion in these methods can cause high-frequency detail loss and image smoothing. To address this, MCDNet [34] employs a multi-scale mechanism and dilated convolutions to extract edge information while using a composite loss function to reduce smoothing. Since conventional CNNs process limited temporal information, LTSC3D [36] introduces a three-dimensional fully convolutional spatiotemporal fusion model based on multidimensional datasets (MDDs). To enhance fusion accuracy in heterogeneous regions, StfNet [39] independently learns spatial and temporal information, leveraging structural similarity and texture features between images. These advancements have refined existing models and addressed spatial and temporal information loss to some extent.

**Figure 6:**
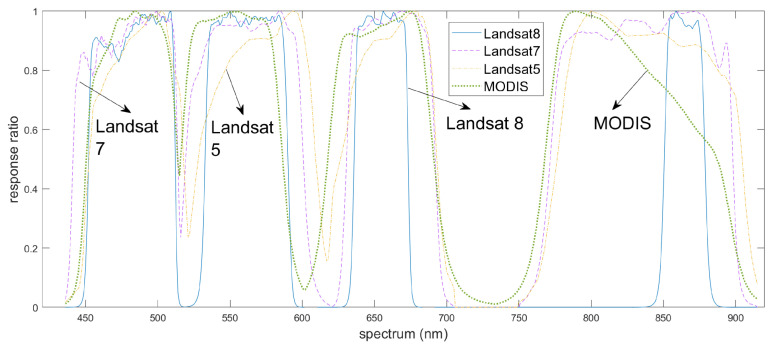
Spectral response functions of the Landsat series and MODIS [84].

Differences between sensors present another challenge for conventional CNN-based spatiotemporal fusion models. Variations in spectral (Figure 6) and geometric characteristics across sensors can introduce biases between images, affecting fusion accuracy. To mitigate this, BiaSTF [43] uses convolutional networks to learn sensor biases, significantly reducing spectral and spatial distortions. Another method, MUSTFN [38], improves performance through multi-level and multi-scale feature extraction. It preserves spatial details and weights neighboring pixels to address information loss caused by Landsat-7 scan-line corrector (SLC) failure and cloud occlusion. By leveraging multi-level feature extraction and automated learning, conventional CNN-based methods can effectively integrate data from different sensors, minimizing sensor discrepancies and enhancing fusion accuracy and stability.

Through the aforementioned studies, conventional CNN-based fusion methods have been extensively explored, prompting researchers to apply them in practical scenarios. To meet application needs, improved spatiotemporal fusion models have been proposed. For example, MSTTIFN [40] enhances fusion for land surface temperature by extracting multi-scale features and texture information, addressing input noise propagation and information loss. CIG-STF [42] integrates change detection with spatiotemporal fusion to improve performance in regions with land-cover changes. These studies show that improved CNN-based fusion methods offer strong adaptability and excellent performance across diverse applications.

Conventional CNN-based methods have advanced in nonlinear mapping, spatial detail reconstruction, and sensor inconsistencies, showing good performance in certain fusion scenarios. However, as spatiotemporal fusion scenarios become more complex and data volumes increase, these methods still have drawbacks in capturing details and handling diverse applications. To address this, residual-based CNN methods have been developed, improving feature extraction and spatiotemporal fusion accuracy with deeper networks and skip connections.

#### 2.1.2. Residual-Based CNN Methods

**Figure 7:**
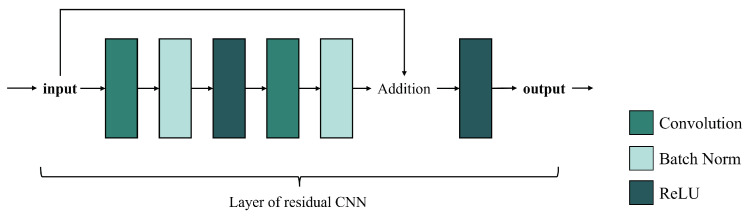
Structure of a typical residual block.

Residual-based CNN methods refer to convolutional neural networks with residual blocks. Originally proposed by [31], residual blocks are designed to create skip connections between different layers of a CNN, which solves the problem of gradient vanishing and model degradation in deep networks. As shown in Figure 7, residual connections add the input to the output of the subsequent layers, allowing gradients to propagate directly to previous layers. This structure helps the network learn identity mappings, increases depth while maintaining efficient training, and improves model expressiveness and generalization.

**Figure 8:**
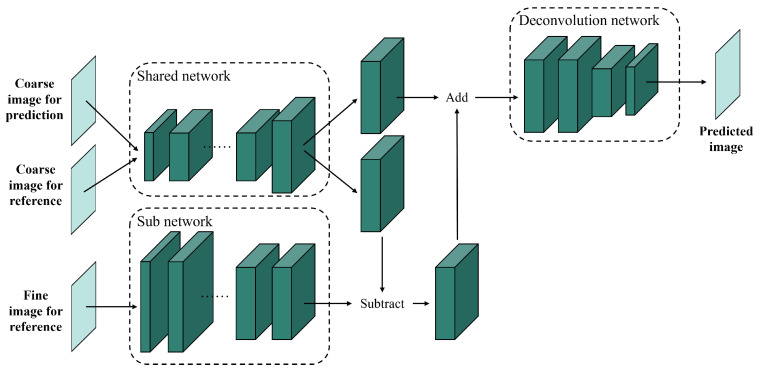
Architecture of DCSTFN [26].

Similar to conventional CNN-based models, residual-based CNN models aim to tackle the shortcomings of traditional spatiotemporal fusion methods. In 2018, Tan et al. [26] proposed the first residual-based CNN fusion network, DCSTFN, which uses residual blocks to find a direct nonlinear mapping between coarse and fine images. The architecture of DCSTFN includes three parts, as shown in Figure 8: first, a shared network expands coarse images; then, a sub-network extracts features from fine images; finally, the extracted features of the fine and coarse images are fused using deconvolution. These convolutional and deconvolutional layers greatly improve the accuracy and robustness of spatiotemporal fusion. However, DCSTFN still has flaws. The use of the mean squared error (MSE) as a loss function often leads to blurred predictions. To address this, the enhanced DCSTFN (EDCSTFN) [45] introduces a composite loss function that preserves high-frequency information and reduces blurriness. In EDCSTFN, spectral information is derived from spectrum changes between the reference and prediction dates. Additionally, to reduce information loss from the direct summation of feature maps in DCSTFN, DMNet [49] employs skip connections within a multi-scale feature extraction framework to preserve temporal variations and spatial details. To handle large variations in spatial resolution, MSISR-STF [85] integrates Graph Neural Networks (GNNs) with residual convolutional networks to find similar pixels between coarse and fine images, aggregating them into graph-structured information to enhance the super-resolution process. By integrating skip connections, residual-based CNN methods have successfully addressed blurriness and information loss in traditional approaches.

In addition to enhancing traditional spatiotemporal fusion methods, residual-based CNN fusion models have significantly improved conventional approaches like STFDCNN, StfNet, and DL-SDFM. For instance, Li et al. [47] introduced a residual CNN model in STFDCNN to reduce redundant computations by merging two transitional images. Peng et al. [51] applied residual blocks in STF3DCNN to optimize data structures and improve computational efficiency for long time-series fusion. To address StfNet’s issue of global fusion parameters failing to capture local variations, STFMCNN [67] incorporated a multi-scale two-stream residual network, enhancing local change feature extraction. Moreover, ResStf [53] improved StfNet by using skip connections and a single image pair for spatiotemporal fusion, solving the challenge of obtaining suitable image pairs. In response to DL-SDFM’s limitations in detecting phenological changes, HDLSFM [55] applied a super-resolution residual network to process both phenological and land-cover changes. Furthermore, to mitigate blurriness and high computational costs in CNN methods, residual-based techniques like STFRDN [60], STFDSC [44], and a dual-branch network [46] integrate dense residual blocks, depthwise separable convolution, and a selection kernel mechanism, respectively. These advancements have significantly improved CNN fusion models by optimizing computational efficiency, enhancing local feature extraction, simplifying input requirements, addressing blurriness, and reducing computational intensity.

**Figure 9:**
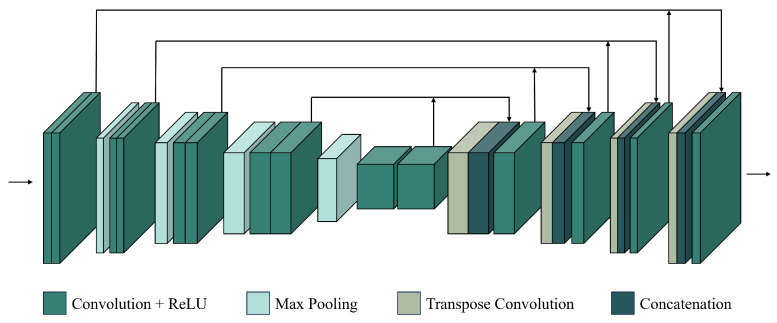
A typical U-Net architecture.

With the development of residual-based CNN fusion methods, spatial information extraction has significantly improved. As a result, some studies now focus on enhancing temporal information extraction in CNN models. Advancements in residual-based CNNs have encouraged researchers to explore their potential in addressing temporal feature extraction challenges in spatiotemporal fusion. In the fusion model by Hoque et al. [48], a U-Net [86] architecture with residual blocks (Figure 9) enhances temporal feature extraction. Most spatiotemporal fusion methods require the reference and predicted dates to be close, but this is difficult due to cloud cover or rain. To address this, Jia et al. [50] applied a temporal constraint mechanism to a residual convolutional fusion model, accounting for differences between the reference and predicted dates. Due to limited temporal information, many fusion methods cannot reconstruct abrupt land-cover changes. Xiong et al. [52] addressed this by using enhanced residual dense networks and modified temporal sequences to reduce reflectance differences and improve prediction accuracy. The introduction of residual CNNs has strengthened the ability of spatiotemporal fusion models to extract temporal information and expanded their application across various data types.

In recent years, increases in the volume and resolution of remote sensing image data have introduced new challenges to spatiotemporal fusion at the data level. Residual-based CNN methods are applied to address feature degradation and limited generalization in different sensor combinations. Image pairs from Landsat and MODIS are commonly used in spatiotemporal fusion studies, but the large spatial resolution difference often leads to feature degradation. To solve this, TSDTSF [54] improves coarse image features using residual convolution and feature transformation, while DPSTFN [56] adopts a progressive fusion scheme to enhance MODIS data resolution. Models trained with Landsat and MODIS pairs often generalize poorly to other satellite data. To address this, Htitiou et al. [57] and Wei et al. [58] developed residual-based fusion models using Landsat-8 and Sentinel-2 pairs, and PMS and WFV pairs from GF-1, respectively. The residual convolutional models in [62,64] utilize image pairs from Luojia-01 and VIIRS DNB nighttime sensors, as well as high-resolution PlanetScope and UAV sensors. These residual-based CNN methods have enhanced feature quality and generalization in spatiotemporal fusion models across different sensors, establishing a foundation for applying residual convolution methods in various scenarios.

Studies have shown significant improvements in residual-based CNN fusion models for spatial and temporal information extraction and data processing. These advancements highlight the potential of residual convolutional methods across various scenarios, driving further research on specific fusion applications. For example, Wei et al. [59] proposed MOST, an image mosaicking method using residual-based CNNs for color adjustment. Fu et al. [66] presented STFNet for tropical cyclone intensity estimation. ACFNet [61] and BASNet [63] are residual-based methods for ice lake extraction and flood classification, respectively. STTFN [65] uses skip connections in convolutional networks to reduce spatial detail loss in surface temperature fusion. These applications demonstrate the broad applicability and potential of residual-based CNN spatiotemporal fusion methods across various fields.

Residual-based CNN models for spatiotemporal fusion have significantly improved feature extraction and generalization performance, leading to widespread application and development across various domains. However, as spatiotemporal fusion demands become more complex and refined, these methods face limitations in capturing long-range dependencies and intricate temporal relationships. To overcome these challenges, researchers have integrated attention mechanisms into CNNs to further enhance the accuracy of spatiotemporal fusion models.

#### 2.1.3. Attention-Based CNN Methods

**Figure 10:**
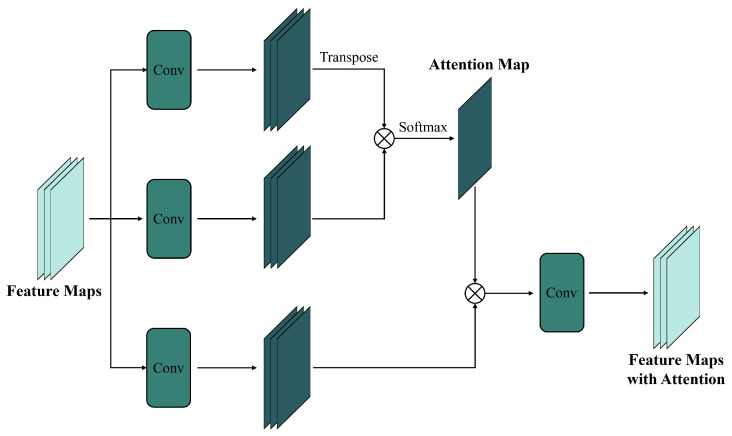
Example of an attention module in a convolutional network (modified from [87]). “Conv” denotes a convolution operation with a kernel size of 1×1.

An attention mechanism [32] enhances a model’s ability to focus on key features or important regions by dynamically assigning weights, improving the efficiency and effectiveness of information extraction. Attention-based convolutional networks (Figure 10) combine the strengths of conventional CNNs and attention mechanisms, adjusting focus to key regions or channels in input images. By introducing spatial or channel attention modules, attention-based CNNs improve performance in various image tasks, including spatiotemporal fusion.

Compared to conventional and residual-based CNNs, attention-based CNNs handle large resolution disparities and complex temporal relationships more effectively, making them key to optimizing fusion performance. For example, PDCNN [72] uses an attention-based pseudo-Siamese network to extract features from both high- and low-resolution images. Sun et al. [74] replaced traditional pixel similarity measurements with a learnable attention module to better utilize input image pairs. Ran et al. [77] introduced SIFnet, an attention-based model that captures resolution differences. STF-EGFA [76] incorporates an edge feature extraction module with attention to refine feature alignment. SCRnet [78] uses a spatial-channel attention mechanism to optimize feature fusion. MANet [80] addresses missing spatial details with separate sub-networks and a residual channel attention upsampling module. These methods enhance spatiotemporal fusion performance by focusing on critical features.

Attention-based CNN methods excel in handling temporal variations, long-term dependencies, and complex spatiotemporal relationships. Non-attention-based approaches often struggle with low accuracy in regions with significant temporal changes. To address this, AMNet [68] integrates attention and multi-scale mechanisms to better capture temporal variations. ASRCNN [70] and RCAN [71] use attention-based CNNs to improve long-term NDVI reconstruction accuracy in heterogeneous regions. These improvements highlight the superiority of attention-based CNN fusion methods in managing complex spatial and temporal relationships. Consequently, more recent attention-based fusion methods have enhanced their ability to handle complex surface variations across various datasets.

Convolutional networks with attention mechanisms can focus on regions that vary widely between images, giving fusion methods based on attention-based CNNs greater robustness and precision in handling complex terrains and surface features. For example, DSTFN [69] integrates residual dense blocks and attention mechanisms to improve performance in abrupt change scenarios and produce high-resolution time series data. CAFE [73] uses multiple processing units with a cross-attention mechanism to capture temporal variations and spatial information, adapting feature weights from spatial and spectral domains. These advancements significantly enhance the adaptability and accuracy of attention-based CNNs in managing surface changes, improving spatiotemporal fusion models across various image scales and resolutions.

Attention-based CNN fusion methods retain fine details from high-resolution images and large-scale patterns from low-resolution images, which is crucial for fusing data with different resolution scales. For example, ECPW-STFN [79] uses a convolutional attention enhancement module to reduce dependence on the number of input images. DSTFNet [75] introduces an attention-driven dual-branch network, where the spatial branch extracts scale information. RCAN-FSDAF [81] integrates attention mechanisms with traditional fusion methods to correct spatial discrepancies between images of different resolutions. These studies improve the adaptability and accuracy of attention-based CNN fusion techniques across various resolutions and image scales.

Attention-based CNN techniques have greatly enhanced the efficiency and versatility of spatiotemporal fusion models by refining feature extraction and fusion in complex scenarios, such as varying resolutions, temporal fluctuations, and land-cover discrepancies. However, despite overcoming some challenges faced by traditional CNNs, they continue to struggle to produce high-quality spatiotemporal fusion images. As a result, fusion techniques using generative adversarial networks have gained significant attention in recent spatiotemporal fusion research.

### 2.2. GAN-Based Fusion Methods

**Figure 11:**
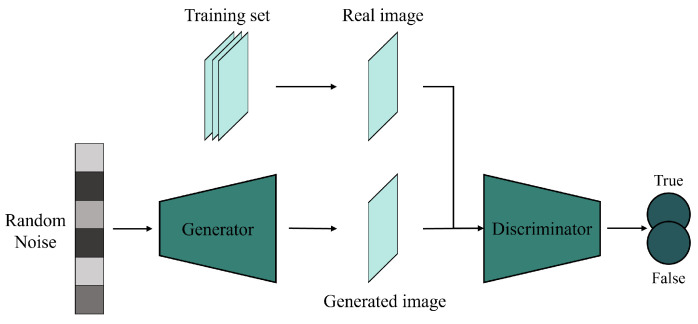
A typical structure of a GAN.

A generative adversarial network (GAN), originally proposed by [22], is a generative model initially used for image generation, denoising, restoration, and conversion. A GAN consists of a generator and a discriminator (Figure 11), where the generator learns to produce realistic data, while the discriminator differentiates between generated and real data. The training process is a two-person zero-sum game [27], where both the generator and discriminator improve simultaneously. When the generator produces data indistinguishable from real samples and the discriminator can no longer differentiate, the network is considered well trained.

Convolutional fusion methods still face challenges like overfitting and poor noise management, which stem from the inherent limitations of convolutional networks. These issues can be addressed with unsupervised learning models like Autoencoders, which are effective in pan-sharpening [88] and have been applied to spatiotemporal fusion. For instance, Chen et al. [67] proposed a conditional Variational Autoencoder-based model for better feature extraction and dimensionality reduction. However, Autoencoders can suffer from blurring effects due to their deterministic encoding-decoding processes. To overcome these limitations, recent research has introduced GAN-based fusion methods, which use adversarial training to improve robustness and generate high-precision fusion images, even from sparse or missing data. The generative mechanism of GANs provides strong generalization abilities, reducing overfitting and data distribution issues. Compared to CNN-based methods, GAN-based approaches yield better fusion outcomes with limited data, enhancing adaptability and generalizability in spatiotemporal fusion. GAN-based spatiotemporal fusion methods are summarized in Table 2.

GAN-based fusion methods were initially designed to enhance the fusion accuracy of CNN-based models by improving feature fusion effectiveness and image generation quality. For example, convolutional fusion methods like STFDCNN and StfNet require separate feature extraction and fusion processes, increasing complexity. To address this, STFGAN [94] introduces an end-to-end adversarial generative network framework, enhancing fusion efficiency through generator and discriminator optimization. PSTAF-GAN [100] combines GANs with attention mechanisms to integrate feature extraction and fusion, improving efficiency and accuracy. CNN-based methods often overlook sensor discrepancies, so SSTSTF [91] incorporates a modular GAN that accounts for spectral, spatial, and sensor differences. Additionally, SMPG [92] integrates a pixel-matching module to address vanishing gradients and insufficient training data. By refining CNN-based models with GANs, these methods improve fusion quality and efficiency while reducing data dependence.

**Figure 12:**
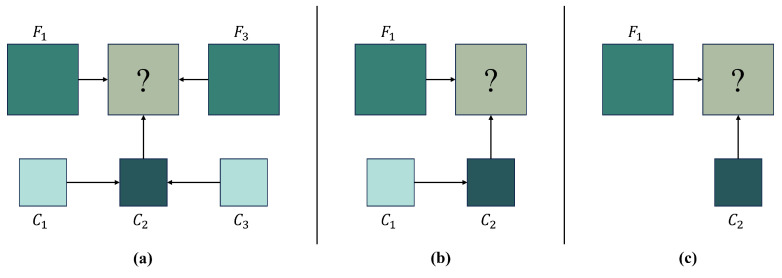
Three types of input schemes in spatiotemporal fusion. (**a**) Five-image input scheme (e.g., STARFM [12]). (**b**) Three-image input scheme (e.g., DCSTFN [26]). (**c**) Two-image input scheme (e.g., GANSTFM [27]). “F” and “C” represent fine images and coarse images, respectively. Subscripts “1”, “3”, and “2” represent the reference dates $t_{1}$, $t_{3}$ and the predicted date $t_{2}$, respectively. “?” represents the predicted image.

Another approach to reducing data dependence is minimizing the number of required inputs in spatiotemporal fusion models. Most traditional fusion models require at least three images and impose strict quality standards on reference images, limiting the broad applicability of spatiotemporal fusion. To address this, GAN-based methods focus on reducing reliance on both the quantity and quality of input images. For example, Tan et al. [27] proposed the GANSTFM model, which uses fine images as conditional inputs and requires only one pair of images, improving the flexibility of spatiotemporal fusion compared to methods that require three or five images (Figure 12). Inspired by GANSTFM, recent studies have developed models like GASTFM [104], which also uses just one pair of images, and TLSRSTF [96], which integrates a mid-resolution image transition module to extract spatial information with fewer inputs.

However, fusion methods using fewer images as input may also overlook surface changes in near-real-time monitoring. To address this, OPGAN [108] enhances the temporal change recognition capability of single-pair fusion models by incorporating temporal variations from different time points. Resolution differences between input images also pose a challenge in traditional fusion methods, where significant disparities hinder spatial information extraction from coarse images. CycleGAN-STF [89] addresses this issue by improving spatial information extraction with a cycle-generative adversarial network and an enhanced loss function. In response to the need for high-resolution spatiotemporal fusion, Liu et al. [103] proposed the StarFusion model, combining traditional methods with super-resolution GANs to merge medium- and high-resolution images. To reduce errors and increase robustness, RSFN [90] improves fusion quality by filtering input noise. Through adversarial training, these GAN-based methods have significantly enhanced spatiotemporal fusion effectiveness in scenarios with limited input data and noise interference.

Nevertheless, current GAN-based methods have certain drawbacks, and the following studies aim to address these issues. For example, to reduce image stitching seams in GANSTFM, Weng et al. [107] proposed an improved method. In response to sensor errors affecting the fusion results in GANSTFM, Wu et al. [99] introduced EDRGAN-STF, which uses degraded resolution versions of input images to rectify the fusion model. To better balance spatial and temporal feature extraction, MCBAM-GAN [93] incorporates multi-level feature extraction, fusion, and multi-scale reconstruction into GAN-based models. Meanwhile, MLFF-GAN [106] enhances sensor discrepancy processing with multi-layered feature extraction techniques. Additionally, to address the loss of spatial details and image blurring caused by neglecting shallow or low-dimensional features, AMS-STF [95] adopts an adaptive multi-scale pyramid network for better feature recognition at different scales. These advancements have greatly improved the performance of GAN-based fusion models in feature extraction, image generation, and handling sensor errors.

Some studies have contributed to the refinement and expansion of GAN-based spatiotemporal fusion methods. For example, Jiang et al. [98] introduced DRCGAN, a GAN-based model for fusing optical and radar images. MOSTGAN [102], which is based on MOST, uses GANs for color adjustment in image stitching. DSFN [97] utilizes GANs for spatiotemporal fusion of land-surface temperature. Additionally, several studies have applied GANs to spatial–spectral–temporal fusion [101,105]. These methods have demonstrated the significant flexibility and potential of GAN-based approaches in addressing various complex tasks across different domains.

Compared to CNN-based methods, spatiotemporal fusion models based on generative adversarial networks (GANs) have significantly enhanced image generation quality and feature fusion effectiveness. However, as the demand for different fusion scenarios grows and data complexity increases, GAN-based methods still encounter challenges related to stability and long-term dependencies. This has led to a growing interest in Transformer-based spatiotemporal fusion models, which have emerged as a promising new research direction in the field, offering the potential for improved handling of long-range dependencies and complex data relationships.

### 2.3. Transformer-Based Fusion Methods

**Figure 13:**
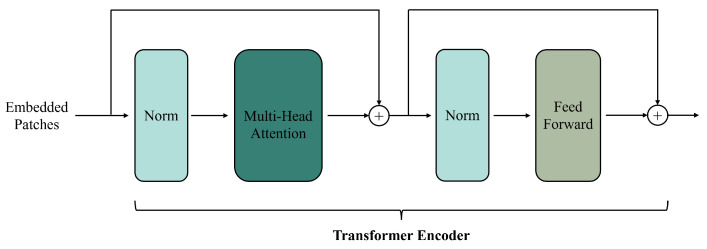
Structure of a Transformer encoder in a Vision Transformer.

Transformers [23], originally developed for natural language processing, use a self-attention mechanism and an encoder–decoder architecture (Figure 13) to capture long-range dependencies and improve training efficiency. Their multi-head attention extracts multiple feature layers. Adapted for computer vision tasks [109], Transformers are widely used in spatiotemporal fusion due to their flexibility and expressiveness.

Handling time-series data has been a challenge for many deep learning-based fusion methods. While Recurrent Neural Networks (RNNs), such as LSTM and GRUs, are commonly used for spatiotemporal fusion [82,83], they struggle with capturing long-range dependencies and suffer from inefficiencies in sequential processing. Transformer-based methods were introduced to overcome these limitations, utilizing self-attention and parallel computation to capture long-term relationships and improve training efficiency.

Transformers offer several advantages over CNNs and GANs. For example, GANs suffer from issues like vanishing gradients and mode collapse [110], and their generators often focus too much on local features, making it difficult to capture long-term dependencies across time or space. In contrast, Transformer-based spatiotemporal fusion models address these issues by extending temporal dependencies and ensuring more stable training. With the help of multi-head attention, Transformers excel at capturing long-term relationships, improving overall training stability. Transformer-based fusion methods are summarized in Table 3.

Transformer-based fusion methods have significantly improved processing flexibility, temporal change accuracy, and feature mapping capabilities compared to CNN-based models. CNNs struggle with limited receptive fields, making it difficult to capture global information. For instance, STF-Trans [115] uses a serialized embedding approach of a Transformer and a dual-stream feature extraction framework to better capture deep features. Convolutional models also struggle to capture temporal variations across different spatial scales. STM-STFNet [116] addresses this by employing the Swin Transformer to extract global information and learn temporal change features. Additionally, MSFusion [111] combines Transformer and CNN modules and uses self-attention to capture global change information, improving temporal feature extraction. These advancements demonstrate the effectiveness of Transformer-based spatiotemporal fusion methods in overcoming the limitations of convolutional models.

Transformer-based fusion methods offer several improvements over GAN-based models, particularly in reducing input noise, lowering computational costs, and enhancing channel feature extraction. For instance, DBTT-FM [117] uses a dual-branch Transformer to extract texture features and applies a composite loss function to reduce noise in generated images. MSNet [28] utilizes Transformers to capture local and global temporal changes, minimizing noise by merging coarse and fine features. EMSNet [114] enhances this by using Transformer embedding and dilated convolutions to extract temporal information and reduce the number of input images. Additionally, GAN methods often require high computational resources, which Transformer-based approaches help mitigate. For example, SwinSTFM [112] uses shifted windows and self-attention mechanisms to reduce redundancy and computational costs. Furthermore, SMSTFM [113] improves feature extraction by incorporating multi-band fusion and three-dimensional convolutions, capturing both spatial and spectral features more effectively. These advancements show that Transformer-based methods outperform GAN-based models while minimizing hardware requirements, thus enhancing the efficiency and adaptability of spatiotemporal data processing.

Transformer-based fusion methods offer significant improvements in flexibility, accuracy in capturing temporal variations, and the ability to map global features. They have successfully addressed challenges such as reducing input noise, lowering computational costs, and enhancing feature extraction compared to CNN- and GAN-based methods. However, research on Transformers for spatiotemporal fusion remains limited, indicating substantial potential for further advancement. Future studies should focus on exploring Transformer models in various spatiotemporal contexts and refining their architectures to better harness their capabilities for handling complex spatiotemporal data.

### 2.4. Diffusion-Based Fusion Methods

**Figure 14:**
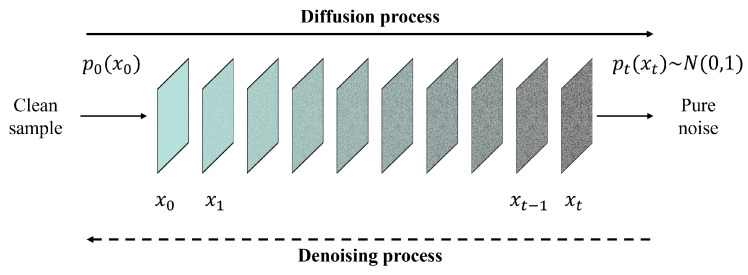
A typical process of a diffusion model.

Recently, diffusion models have gained attention in computer vision [118], particularly Denoising Diffusion Probabilistic Models (DDPMs) [24]. A diffusion model is a generative model that gradually adds random noise to data through a diffusion Markov process. Its training involves two phases: a diffusion process and a denoising process (Figure 14). In the diffusion process, noise is progressively added to real data samples, while in the denoising process, pure noise is gradually removed to recover the original data. By alternating between these two stages, diffusion models learn to generate data that closely resemble the original samples. Given their strong generative capabilities, diffusion models have been applied to various image tasks, such as super-resolution [119], image denoising [120], and image restoration [121]. As a result, some studies have started exploring diffusion models for spatiotemporal fusion, as shown in Table 4.

Huang et al. introduced a diffusion model into the field of spatiotemporal fusion and proposed STFDiff [29]. In response to the issues of spatial, spectral, and temporal uncertainties in current deep learning-based fusion methods and the problem of mode collapse in GAN-based fusion models, STFDiff integrates a diffusion model and a dual-stream U-Net to better predict noise in each time step. The process of spatiotemporal fusion is regarded as a conditional diffusion process in STFDiff, where fine images from reference dates and coarse images from predicted dates serve as the conditional input, while the target images serve as the original input. Experiments with STFDiff have shown that this diffusion-based fusion method outperforms others based on CNNs, GANs, and Transformers, demonstrating great potential for applications. To address sensor and scale errors in existing spatiotemporal fusion methods, Ma et al. utilized the concept of the conditional diffusion model and proposed DiffSTF [122]. DiffSTF takes the structural information from fine images in reference dates and the spectral information from coarse images in predicted dates as the dual conditions for training. Similar to DiffSTF, Wei et al. [123] proposed a diffusion-based fusion method, DiffSTSF, to blend images from GF-1 2-meter panchromatic, 8-meter multispectral and 16-meter wide-field cameras. To improve the fusion results from previous work [58], a multi-conditional diffusion model was utilized to achieve better results than existing CNN-based methods. In DiffSTSF, the diffusion process is regarded as a degradation process that models the downscaling explicitly, while the backward denoise process is considered the fusion process.

These studies demonstrate the improvements and advancements of diffusion-based methods over current deep learning-based models, as well as their great potential in the field of spatiotemporal fusion. However, existing research on diffusion-based fusion models remains lacking. Therefore, future studies should focus on further improving the performance and applicability of diffusion models to maximize their potential in spatiotemporal fusion.

## 3. Evaluations and Applications

Deep learning-based spatiotemporal fusion methods are applied in fields like crop classification, land-cover mapping, and change detection. Comparing their performance and efficiency is crucial for selecting the right model for specific needs. This section explores their applications, compares their performance, and evaluates their adaptability.

### 3.1. Method Comparisons

Evaluating spatiotemporal fusion methods is crucial to determine their effectiveness in remote sensing applications. This assessment focuses on performance, which examines the accuracy and reliability of fused results, and on computational efficiency, which considers processing time and resource use. A comprehensive comparison of these aspects helps identify the best method for specific needs. In this section, we compare CNN-based, GAN-based, Transformer-based, and diffusion model-based fusion methods to assess their accuracy and efficiency.

#### 3.1.1. Performance Evaluation

Performance evaluation is essential to assess how well spatiotemporal fusion methods preserve spatial, temporal, and spectral details. Since the effectiveness of deep learning models can vary based on parameters, training times, and hardware, we conducted a statistical analysis of the fusion performance metrics of four open-source spatiotemporal fusion models (Table 5) using publicly available datasets for an objective comparison.

**Figure 15:**
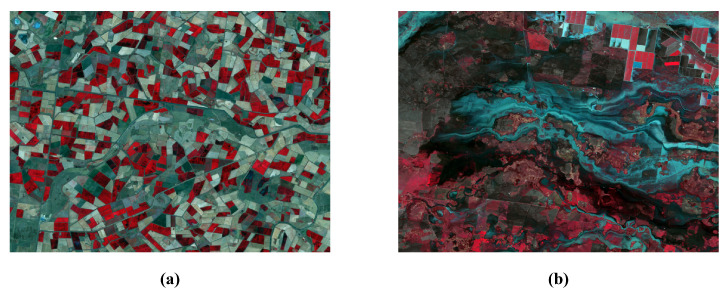
Examples of cropland areas from the CIA dataset (**a**) and a flood event from the LGC dataset (**b**).

We compared the performance of the CNN-based, GAN-based, Transformer-based, and diffusion-based fusion models using two publicly available datasets and five evaluation metrics. The Coleambally Irrigation Area (CIA) dataset pertains to southern New South Wales (NSW, Australia; 145.0675° S, 34.0034° E) and consists of Landsat-7 and MODIS image pairs, capturing phenological changes over a season. The Lower Gwydir Catchment (LGC) dataset pertains to northern NSW (29.0855° S, 149.2815° E) and includes Landsat-5 and MODIS pairs, covering crop growth cycles and a flood event. Examples of cropland areas from the CIA dataset and a flood event from the LGC dataset are shown in Figure 15. The evaluation metrics include the root mean square error (RMSE), structural similarity index (SSIM), and correlation coefficient (CC) for spatial accuracy, as well as the spectral angle mapper (SAM) and relative dimensionless global error in synthesis (ERGAS) for spectral accuracy. The RMSE measures the difference between the predicted and reference values, the SSIM evaluates the structural similarity, the CC assesses the linear relationship between the predicted and reference images, the SAM measures the spectral similarity, and the ERGAS evaluates the overall spectral quality. Note that since STFDiff appears only once in the literature, its box plot has no height.

**Figure 16:**
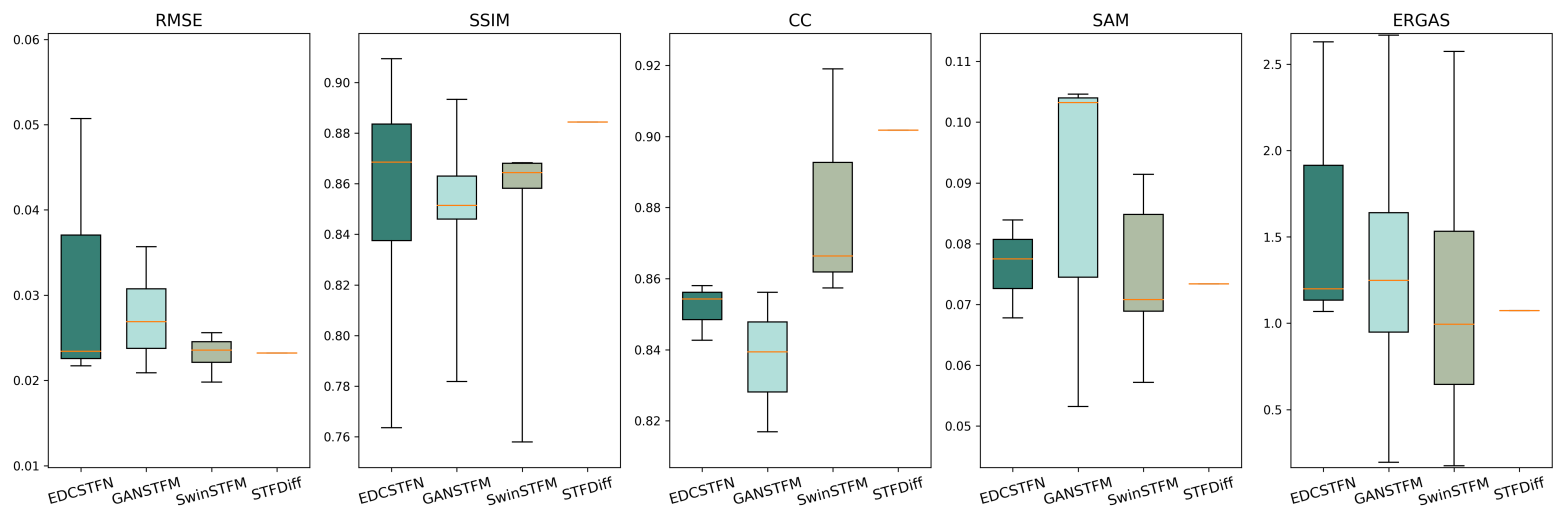
Box plots for quantitative performance evaluation on the CIA dataset.

As shown in Table 6 and Figure 16, the RMSE decreases consistently across the CIA dataset as the spatiotemporal fusion models advance from CNN to diffusion, highlighting that the reduction in the pixel-level RMSE is a key focus of these models. However, significant fluctuations in the SSIM suggest that these models struggle with global representation. For the correlation coefficient (CC), the Transformer and diffusion models outperform the CNN and GAN models, demonstrating that more complex models better preserve spatiotemporal correlations by learning intricate nonlinear mappings. In terms of the SAM, the Transformer and diffusion models show greater advantages. However, the performance of the ERGAS fluctuates for all models, likely due to deep learning models using small data blocks for training, limiting their ability to leverage global information. The CNN model, with its limited receptive field, shows poorer ERGAS performance.

**Figure 17:**
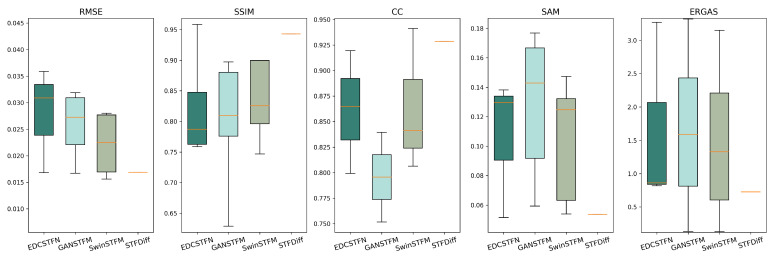
Box plots for quantitative performance evaluation on the LGC dataset.

Similar to the CIA dataset, the indicators in the LGC dataset reflect the performance of the four models, as shown in Table 7 and Figure 17. The diffusion-based model consistently outperforms the others in all aspects. In terms of the RMSE and SSIM, the more advanced models show better performance. However, the correlation coefficient (CC) for the GAN-based model is lower than that of the others, possibly due to the GAN’s emphasis on the adversarial process, which may neglect learning correlations between predicted and real images, leading to texture and detail discrepancies. In contrast, the diffusion-based method improves the CC by more effectively learning the data distribution. Regarding the SAM and ERGAS, all models exhibit significant fluctuations, similar to their performance on the CIA dataset.

#### 3.1.2. Computational Efficiency

Computational efficiency is crucial for the practical deployment of spatiotemporal fusion methods, especially in large-scale remote sensing tasks that require processing vast amounts of high-resolution data. In deep learning, the size of model parameters is the primary factor affecting training and inference time, as seen in Table 8. CNN-based models are mainly affected by the number of layers and the size of convolutional kernels. GAN-based methods, due to simultaneous training of the generator and discriminator, require longer training times compared to CNN-based methods. Transformer models, due to the self-attention mechanism, consume large amounts of memory and require longer training times. Diffusion models, which require thousands of diffusion steps for each image, significantly increase training time.

Additionally, training time is influenced by the number of iterations required for model convergence. CNN-based methods, with their local receptive fields and shared weights, generally have shorter training times. In contrast, Transformer, GAN, and diffusion models have slower convergence speeds. GAN-based models experience slower convergence due to the instability in adversarial training, while diffusion models also suffer from slower loss reduction due to the iterative nature of the diffusion process. These factors contribute to the extended training times of GAN and diffusion models.

### 3.2. Model Applicability

Model applicability examines the suitability of deep learning-based fusion models across various scenarios and datasets. This section assesses the adaptability of CNN, GAN, Transformer, and diffusion models through quantitative analysis of their evaluation metrics. It also evaluates their feasibility in different tasks and conditions, considering how data heterogeneity (such as variations in data types, quality, or resolution) impacts their performance. This analysis helps determine which models are best suited for specific spatiotemporal fusion applications and the challenges posed by different real-world data scenarios.

#### 3.2.1. Feasibility for Different Scenarios

For the CIA dataset, which features small farmland areas with notable spatial heterogeneity over a single growing season, deep learning-based fusion methods face challenges related to both spatial and temporal variations. CNN models are less suitable due to their focus on local spatial patterns, which may not be sufficient to capture the dataset’s high spatial diversity. While CNNs can extract texture and spectral features, they struggle to model complex relationships between varying crop types and environmental factors. GAN models are more appropriate for this dataset, as they excel at synthesizing spatial patterns, which helps capture the diversity of conditions in the farmland regions. Transformer models are highly effective due to their ability to model long-range dependencies across both spatial and temporal dimensions, addressing spatial heterogeneity and complex relationships in crop dynamics. Diffusion models, while computationally intensive, can enhance spatial feature generation and handle heterogeneity through their iterative refinement process, although they may struggle to capture the rapid changes in crop dynamics during the season.

For the LGC dataset, which spans an entire year of crop growth with significant temporal changes, including a flood event in a mountainous region, deep learning models face unique challenges. CNN models are limited due to their inability to capture long-range temporal dependencies and complex spatial patterns, especially in areas with large elevation changes and extreme events like floods. GAN models can generate high-resolution images but struggle to accurately model temporal sequences, particularly during abrupt environmental changes. Transformer models excel in this scenario, as their ability to capture long-range temporal dependencies allows them to effectively model the entire crop growth cycle and extreme events. Although computationally intensive, diffusion models are highly effective for this dataset, as they can iteratively refine image quality and handle the complex spatiotemporal variations caused by the mountainous terrain and flood events.

#### 3.2.2. Impact of Data Heterogeneity

The CIA dataset is marked by significant data heterogeneity, with variations in crop types, soil conditions, and farming practices across small farmland regions. This introduces considerable spatial and temporal variability, which is challenging for deep learning models. CNN-based methods struggle with this high heterogeneity because they are limited in their ability to capture long-range spatial dependencies, making them less effective in diverse environments with significant land-cover variation. GANs also face difficulties in modeling the complex temporal variations in crop growth across different farming regions, and their inability to capture long-term dependencies further limits their performance in such settings. Transformer models, however, are more capable of handling this heterogeneity due to their ability to model complex relationships between different crop types, seasonal changes, and environmental factors over time. Diffusion models, with their iterative refinement process, also show promise in addressing data heterogeneity. They progressively refine predictions while preserving key spatial features, improving the robustness of the model, and helping to mitigate inconsistencies caused by the dataset’s heterogeneous conditions.

### 3.3. Practical Applications

**Figure 18:**
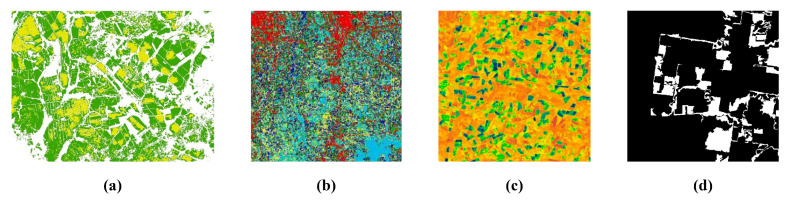
Application examples of spatiotemporal fusion. (**a**) Crop classification. (**b**) Land-cover classification. (**c**) Vegetation monitoring. (**d**) Change detection.

Spatiotemporal fusion techniques based on deep learning have been increasingly utilized to overcome the limitations of traditional remote sensing data, offering improved spatial and temporal resolutions for a wide range of applications. In particular, applications in crop classification, land-cover classification, vegetation monitoring, and change detection have benefited from deep learning-based methods (Figure 18). These applications provide valuable insights for environmental management and agricultural planning.

#### 3.3.1. Crop Classification

In crop classification, mixed pixels in low-resolution images often blur the spectral characteristics of different land-cover types, leading to lower classification accuracy. Time-series remote sensing datasets generated through spatiotemporal fusion help monitor high-frequency changes, offering advantages in crop classification. For example, Zhan et al. [83] proposed the CNN-based TSSTFN fusion model to generate multi-temporal high-resolution NDVI, improving classification accuracy between soybean and corn. Their experiments showed a significant improvement in the kappa coefficient, rising from 69.2% before fusion to 74.22–82.44% after fusion. This highlights that the construction of high-resolution time-series NDVI through CNN-based fusion methods plays a crucial role in improving crop classification accuracy by leveraging the spectral and textural features of remote sensing images. CNNs are well suited for capturing local spatial features such as shape, texture, and spectral patterns, making them effective in distinguishing different crop types. Additionally, the relatively simple architecture and fast training speed of CNNs make them efficient for processing large-scale remote sensing datasets with reduced computational costs.

#### 3.3.2. Land-Cover Classification

Land-cover classification, a key component of Earth observation systems, plays an essential role in climate and ecological studies. High-resolution imagery generated by spatiotemporal fusion methods improves land-cover classification accuracy and supports time-series analysis. Similar to crop classification, CNN-based fusion methods have been effectively applied to enhance land-cover classification accuracy across various landscapes. Studies have shown significant improvements in performance with deep learning-based models [79]. For example, AMSDFNet achieved a 2–+3% increase in both overall pixel accuracy (PA) and mean intersection over union (mIOU) compared to milestone methods [124]. DSTFNet improved the F1 score from 0.865 to 0.909 and demonstrated better transferability than direct classification using U-Net [75]. These CNN-based spatiotemporal fusion methods outperform non-fusion methods in land-cover classification, offering advantages for managing the complexity and diversity of land-cover categories. However, for more intricate scenarios like land-cover classification, GAN-based, Transformer-based, and diffusion-based methods are more suitable and generally perform better than CNN-based fusion models [29,105].

#### 3.3.3. Vegetation Monitoring

Dense time-series remote sensing imagery is essential for continuous and reliable phenological monitoring, particularly for tracking growth processes. However, obtaining high-resolution, dense, and cloud-free imagery remains a challenge. To overcome this, spatiotemporal fusion is increasingly applied in vegetation monitoring, with the Normalized Difference Vegetation Index (NDVI) being the most commonly used vegetation index. Many studies utilize spatiotemporal fusion to generate dense time-series NDVI images for long-term, high-resolution vegetation monitoring. CNN-based models typically outperform traditional fusion methods in terms of accuracy, showing improvements in the root mean square error (RMSE) and structural similarity index (SSIM), especially in areas with phenological changes and shadows, like forests. This highlights the robustness of CNNs in spatiotemporal fusion for crop monitoring, both at the pixel and feature levels. Additionally, Transformer-based methods, known for their superior ability to capture temporal dependencies, are also effective for vegetation monitoring, although they incur significant computational costs.

#### 3.3.4. Change Detection

Time-series land-cover imagery is essential for surface change monitoring, as it provides high-resolution images that capture subtle changes in the Earth’s surface. Spatiotemporal fusion techniques that generate dense, high-resolution land-cover time-series imagery play a key role in improving change detection accuracy. For example, the GAN-based fusion method SMPG [92] employs spatiotemporal fusion for change detection in snow areas. By providing detailed spatial and temporal information, high-resolution synthetic images produced through spatiotemporal fusion can significantly enhance change detection accuracy. Experimental results showed that the SMPG method achieved change detection error rates of 0.17% and 0.84% in two regions, outperforming other fusion methods with error rates of 0.68% and 1.89%. The superior performance of GAN-based fusion models in change detection is attributed to their ability to generate more realistic and accurate synthetic images by effectively learning the underlying data distribution, thus improving the detection of subtle surface changes.

## 4. Current Issues and Future Directions

Current studies on deep learning-based spatiotemporal fusion methods have shown considerable progress in feature extraction, spatiotemporal modeling, and computational efficiency. However, several critical challenges persist. Existing models struggle to precisely detect subtle and ephemeral changes. The discrepancies in spectral and spatial alignment among sensors also hinder the precise extraction and integration of image features. Additionally, the absence of standardized benchmark datasets and evaluation metrics impedes the generalization and uniform assessment of these methods. The complexity of deep learning models also results in higher computational costs, and variations in input imagery and network structures introduce uncertainty into existing spatiotemporal fusion models. Therefore, this section discusses the current challenges facing deep learning-based spatiotemporal fusion methods and proposes possible future directions for addressing these issues.

### 4.1. Land-Cover Changes

Despite the strengths of CNNs, GANs, and Transformers in spatiotemporal fusion, they struggle to capture subtle or abrupt land-cover changes. In CNN-based methods, downsampling in convolutional layers can miss small or transient variations, making it difficult to detect changes in small regions. While GANs improve global consistency and fine detail extraction, their generators focus on overall image quality, often neglecting subtle changes. Transformers, although capable of capturing long-term dependencies with self-attention, struggle to focus on small or sudden changes.

Therefore, future deep learning research on spatiotemporal fusion should prioritize identifying small or transient changes by incorporating techniques like multi-scale feature extraction and adaptive attention mechanisms. Multi-scale feature extraction, such as using multi-scale convolutional networks or hierarchical pyramid structures [125], helps models capture changes across various spatial and temporal scales, improving accuracy in detecting subtle variations. This approach allows for precise detection of both local and large-scale changes, making it especially effective in complex and dynamic environments where changes vary in intensity across regions. Additionally, integrating adaptive attention mechanisms offers a promising way to enhance model performance. These mechanisms enable the model to dynamically focus on regions of interest, prioritizing areas with significant or transient changes while minimizing the computational resources spent on less relevant areas. Furthermore, attention mechanisms improve a model’s robustness against noisy or incomplete data and make fusion methods more adaptable to varying complexities in land-cover changes. This can provide more reliable and actionable insights for applications such as land-use change detection, crop monitoring, and climate change assessment. Future research in this area will be crucial for developing more efficient and accurate deep learning-based spatiotemporal fusion models.

### 4.2. Sensor Differences

Sensor differences, including reflectance and geometric registration discrepancies [126], significantly impact the accuracy and reliability of spatiotemporal fusion outcomes. Reflectance inconsistencies arise from variations in bandwidth, spectral response functions, and atmospheric conditions, resulting in different reflectance values for the same surface feature across sensors [127]. Such reflectance discrepancies, especially between coarse- and fine-resolution images, can bias fusion results and degrade their quality. Geometric registration errors, caused by misalignment between corresponding locations, result from differences in sensor viewing angles, swath widths, and geometric correction techniques [128]. In deep learning-based fusion methods, especially CNNs, model performance is heavily reliant on accurate training data, and misregistration between sensor images can cause substantial inaccuracies [35].

Future research on deep learning-based spatiotemporal fusion should focus on minimizing sensor-induced errors and developing models that can effectively address reflectance and spatial registration discrepancies [129]. One promising direction is to design adaptive fusion algorithms that dynamically adjust for sensor-specific discrepancies by learning the sensor-to-sensor mapping during training. Additionally, spatial and spectral alignment methods could be improved by leveraging deep learning models that learn complex geometric transformations. For example, integrating spatial transformer networks (STNs) into spatiotemporal fusion models could enable the models to automatically learn and apply optimal geometric transformations, addressing misalignments caused by satellite or sensor motion, varying pixel sizes, and other registration issues. Another promising approach is to incorporate physical models into deep learning frameworks. By embedding domain-specific physical knowledge into the neural network structure, models could maintain consistency with known physical laws while learning the fusion task. Future studies should focus on enhancing the robustness of deep learning-based fusion models by developing effective algorithms that address sensor discrepancies.

### 4.3. Datasets and Assessment Metrics

Benchmark datasets and evaluation metrics are crucial for advancing and comparing deep learning models. Benchmark datasets are essential for training and validating model performance, with their scale and diversity directly affecting model accuracy [65]. Larger and more varied datasets significantly improve model performance, particularly for complex models with many parameters. Evaluation metrics guide the formulation of loss functions and assess feature extraction and integration capabilities. In deep learning-based fusion methods, these metrics influence the design of loss functions and the training process. Thus, creating standardized benchmark datasets and unified evaluation metrics is key to improving deep learning-based spatiotemporal fusion algorithms and their practical applications.

**Figure 19:**
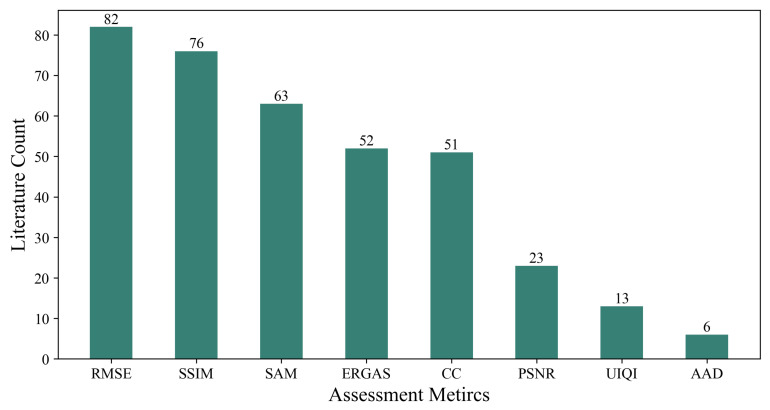
Literature count of various assessment metrics in deep learning-based spatiotemporal fusion methods.

However, existing spatiotemporal fusion studies face challenges due to insufficient benchmark datasets (Table 9) and a lack of unified evaluation metrics (Figure 19). Current research relies on limited and specific datasets, which introduce biases in geographic representation, temporal duration, and sensor types, thus restricting the generalizability and applicability of the models [62]. Additionally, the evaluation metrics used in spatiotemporal fusion research are diverse and cover various dimensions, such as spectral, spatial, and visual quality. This diversity complicates comprehensive evaluations and comparisons of fusion results across studies [130]. For instance, some studies use only the RMSE and r, which measure spectral feature accuracy, neglecting spatial features [131]. Furthermore, redundancy exists in current evaluation metrics; for example, some studies use both the RMSE and AAD, which are highly correlated, leading to unnecessary duplication [130].

Hence, future research should focus on enhancing the diversity and scale of datasets by incorporating a broader range of geographic regions, temporal durations, and sensor types to improve model generalization. Establishing benchmark datasets and standardized evaluation metrics will enhance the accuracy and applicability of future spatiotemporal fusion research. Furthermore, standardized assessment metrics should be developed for comprehensive model evaluation and comparison. For instance, Zhu et al. [130] proposed a novel framework for assessing spatiotemporal fusion performance by incorporating four indicators from both spatial and spectral aspects. Their study also designed a visual polar coordinate chart, enabling cross-comparison of different fusion methods while considering input data and surface features. Additionally, Guo et al. [134] introduced a new evaluation metric, the SSAM, which simultaneously evaluates both the spatial and spectral accuracy of fused imagery. Compared to existing metrics, the SSAM offers a more comprehensive and intuitive evaluation of fusion image quality, thereby facilitating cross-comparison studies of various spatiotemporal fusion methods. Therefore, future studies focusing on benchmark datasets and standardized evaluation metrics will be pivotal in advancing deep learning-based spatiotemporal fusion.

### 4.4. Efficiency and Uncertainty

Deep learning-based fusion methods often involve complex network architectures with a large number of parameters, leading to high computational costs and long training times. CNN- and GAN-based methods typically include hundreds of thousands of parameters, while Transformer models can exceed a million parameters. This limitation affects their feasibility for large-scale and real-time applications [112]. Additionally, these methods are sensitive to input image quality and data noise, which introduces uncertainty and instability into fusion results [27]. The transferability of models to new datasets or different regions remains challenging, as models trained on specific datasets may not generalize well to others due to variations in sensor characteristics and environmental conditions. Furthermore, the repeatability of results can be influenced by factors such as random initialization and hyperparameter tuning, leading to variations in performance across different training runs. These challenges further complicate the deployment of deep learning-based fusion methods in real-world applications.

Future deep learning-based spatiotemporal fusion methods should prioritize improving efficiency through algorithm optimization, model structure enhancements, and parallel computation. One approach is to develop adaptive models that adjust computational requirements based on data quality and input noise. For example, uncertainty-aware networks could be employed to automatically highlight reliable regions of input data while minimizing the influence of noisy or missing areas. Additionally, incremental learning techniques, such as lifelong learning or transfer learning [135], can help models continuously learn from new data without forgetting previous knowledge. Online updating mechanisms [136] could allow models to adapt in real time to incoming data streams. Federated learning approaches might also be beneficial, enabling decentralized models to train across multiple devices or locations, preserving privacy while efficiently processing large, diverse datasets. Furthermore, future innovations should explore model compression and optimization techniques using GPUs or parallel computing to reduce computational costs for practical applications.

## 5. Conclusions

Over the past decade, spatiotemporal fusion techniques for remote sensing images have become relatively mature, and deep learning methods have been extensively applied in this field. Deep learning-based spatiotemporal fusion algorithms demonstrate great advantages in both accuracy and efficiency. Yet, they also face limitations and drawbacks. This paper comprehensively reviews existing deep learning-based fusion methods, categorizing them based on neural network model principles, and outlines the research progress and current trends in this area. Through a detailed analysis of different algorithms, this paper identifies the remaining challenges in deep learning-based spatiotemporal fusion research and provides an outlook on potential future directions. The main contributions of this paper are as follows:

1. This paper provides a detailed classification of existing deep learning-based spatiotemporal fusion methods based on network structures and categorizes them into four main types: convolutional neural network-based methods, generative adversarial network-based methods, Transformer-based methods, and diffusion-based methods. This paper analyzes and compares the different principles, advantages, and disadvantages of each deep learning-based method and outlines the evolution and development of research in this area. As neural network models are increasingly being applied in spatiotemporal fusion, the comprehensive analysis and summary presented in this paper serve as a helpful resource for future research on deep learning-based spatiotemporal fusion methods.
2. This paper provides an in-depth exploration of deep learning-based spatiotemporal fusion methods, presenting application examples, performance evaluations, and method comparisons to assess their effectiveness and computational efficiency. By evaluating four deep learning-based fusion models from CNN-based, GAN-based, Transformer-based, and diffusion-based methods, this paper offers valuable insights into the strengths and limitations of various approaches, considering different scenarios and the impact of data heterogeneity. The analysis highlights the importance of model adaptability, computational efficiency, and robustness to data variations, providing a solid foundation for improving the performance and scalability of deep learning-based spatiotemporal fusion methods.
3. This paper identifies four challenges currently faced in deep learning-based spatiotemporal fusion studies. Difficulties in recognizing land-cover changes and the insufficient consideration of sensor differences are common obstacles for deep learning-based fusion models. The limited data scale, the lack of variety in spatiotemporal fusion datasets, the incompleteness and redundancy of evaluation metrics, and the low computational efficiency and uncertainty of deep learning-based models are important issues that future studies need to tackle. In response to these challenges, this paper proposes several potential solutions and provides useful references for subsequent research and applications of deep learning-based spatiotemporal fusion methods.

## Figures and Tables

**Table 1:** CNN-based spatiotemporal fusion methods.

	Year	Method	Year	Method
Conventional CNN-based	2018	STFDCNN [33]	2021	MCDNet [34]
	2019	VDCN [35]	2022	LTSC3D [36]
	2019	ESRCNN [37]	2022	MUSTFN [38]
	2019	StfNet [39]	2022	MSTTIFN [40]
	2020	DL-SDFM [41]	2024	CIG-STF [42]
	2020	BiaSTF [43]		
Residual CNN-based	2019	DCSTFN [26]	2022	STFDSC [44]
	2019	EDCSTFN [45]	2022	Li et al. [46]
	2019	Li et al. [47]	2022	Hoque et al. [48]
	2020	DMNet [49]	2022	MTDL-STF [50]
	2020	STF3DCNN [51]	2022	ERDN [52]
	2020	ResStf [53]	2022	TSDTSF [54]
	2021	HDLSFM [55]	2022	DPSTFN [56]
	2021	Htitiou et al. [57]	2022	Wei et al. [58]
	2021	MOST [59]	2023	STFRDN [60]
	2021	ACFNet [61]	2023	UAV-Net [62]
	2021	BASNet [63]	2024	Zeng et al. [64]
	2021	STTFN [65]	2024	STFNet [66]
	2022	STFMCNN [67]		
Attentional CNN-based	2021	AMNet [68]	2022	DSTFN [69]
	2021	ASRCNN [70]	2023	RCAN [71]
	2022	PDCNN [72]	2023	CAFE [73]
	2022	SL-STIF [74]	2023	DSTFNet [75]
	2022	STF-EGFA [76]	2024	SIFnet [77]
	2022	SCRnet [78]	2024	ECPW-STFN [79]
	2022	MANet [80]	2024	RCAN-FSDAF [81]

**Table 2:** GAN-based spatiotemporal fusion methods.

Year	Method	Year	Method
2020	CycleGAN -STF [89]	2022	RSFN [90]
2021	SSTSTF [91]	2022	SMPG [92]
2021	GANSTFM [27]	2023	MCBAM-GAN [93]
2021	STFGAN [94]	2023	AMS-STF [95]
2021	TLSRSTF [96]	2023	DSFN [97]
2022	DRCGAN [98]	2023	EDRGAN-STF [99]
2022	PSTAF-GAN [100]	2023	BPF-MGAN [101]
2022	MOSTGAN [102]	2024	StarFusion [103]
2022	GASTFN [104]	2024	Sun et al. [105]
2022	MLFF-GAN [106]	2024	Weng et al. [107]
2022	OPGAN [108]		

**Table 3:** Transformer-based spatiotemporal fusion methods.

Year	Method	Year	Method
2021	MSNet [28]	2022	MSFusion [111]
2022	SwinSTFM [112]	2023	SMSTFM [113]
2022	EMSNet [114]	2024	STF-Trans [115]
2022	DBTT-FM [100]	2024	STM-STFNet [116]

**Table 4:** Diffusion-based fusion methods.

Year	Method
2024	STFDiff [29]
2024	DiffSTF [122]
2024	DiffSTSF [123]

**Table 5:** Open-source methods.

Type	Method	Link
CNN-based	EDCSTFN	https://github.com/theonegis/edcstfn (accessed on 11 February 2025)
GAN-based	GANSTFM	https://github.com/theonegis/ganstfm (accessed on 11 February 2025)
Transformer-based	SwinSTFM	https://github.com/LouisChen0104/swinstfm (accessed on 11 February 2025)
Diffusion-based	STFDiff	https://github.com/prowDIY/STF (accessed on 11 February 2025)

**Table 6:** Quantitative performance evaluation on the CIA dataset. Values in bold represent the model with the optimal average performance.

		EDCSTFN	GANSTFM	SwinSTFM	STFDiff
RMSE	Min	0.0217	0.0209	0.0198	0.0232
	Max	0.0507	0.0357	0.0256	
	**Avg**	0.0319	0.0276	**0.0231**	
SSIM	Min	0.7936	0.7818	0.7579	0.8844
	Max	0.9094	0.8933	0.8683	
	**Avg**	0.8525	0.8471	**0.8434**	
CC	Min	0.8427	0.8169	0.8574	0.9018
	Max	0.8580	0.8562	0.9190	
	**Avg**	0.8517	**0.8375**	0.8809	
SAM	Min	0.0678	0.0532	0.0572	**0.0734**
	Max	0.0839	0.1046	0.0914	
	**Avg**	0.0764	0.0879	0.0746	
ERGAS	Min	1.0677	0.1955	0.1754	**1.0732**
	Max	2.6280	2.6675	2.5728	
	**Avg**	1.6315	1.3399	1.1842	

**Table 7:** Quantitative performance evaluation on the LGC dataset. Values in bold represent the model with the optimal average performance.

		EDCSTFN	GANSTFM	SwinSTFM	STFDiff
RMSE	Min	0.0168	0.0167	0.0174	**0.0169**
	Max	0.0359	0.0319	0.0280	
	**Avg**	0.0279	0.0258	0.0222	
SSIM	Min	0.7585	0.6290	0.7470	0.9429
	Max	0.9585	0.8972	0.8997	
	**Avg**	0.8228	**0.7984**	0.8336	
CC	Min	0.7993	0.7517	0.8065	0.9286
	Max	0.9195	0.8395	0.9412	
	**Avg**	0.8612	**0.7956**	0.8630	
SAM	Min	0.0515	0.0593	0.0539	**0.0536**
	Max	0.1382	0.1769	0.1474	
	**Avg**	0.1064	0.1275	0.1043	
ERGAS	Min	0.8180	0.1336	0.1310	**0.7258**
	Max	3.2709	3.3205	3.1502	
	**Avg**	1.6504	1.6581	1.4844	

**Table 8:** Parameters for each model.

Method	Parameters
EDCSTFN	280,000
GANSTFM	4,180,000
SwinSTFM	39,665,893
STFDiff	4,590,000

**Table 9:** Current open-source spatiotemporal fusion datasets. “Citations” indicate references within deep learning spatiotemporal fusion methods (as of 12 December 2024).

Datasets	Year	Data Source	Citations
CIA [132]	2013	Landsat-7|MODIS	77
LGC [132]		Landsat-5|MODIS	73
AHB [133]	2020	Landsat-8|MODIS	12
DX [133]			6
TJ [133]			5

## Data Availability

Not applicable.

## References

[1] Mewes B., Schumann A.H. (2019). An agent-based extension for object-based image analysis for the delineation of irrigated agriculture from remote sensing data. Int. J. Remote Sens..

[2] Sun Y., Luo J., Xia L., Wu T., Gao L., Dong W., Hu X., Hai Y. (2020). Geo-parcel-based crop classification in very-high-resolution images via hierarchical perception. Int. J. Remote Sens..

[3] Aneece I., Thenkabail P.S., McCormick R., Alifu H., Foley D., Oliphant A.J., Teluguntla P. (2024). Machine Learning and New-Generation Spaceborne Hyperspectral Data Advance Crop Type Mapping. Photogramm. Eng. Remote Sens..

[4] Liang L., Tan B., Li S., Kang Z., Liu X., Wang L. (2022). Identifying the Driving Factors of Urban Land Surface Temperature. Photogramm. Eng. Remote Sens..

[5] Al-Doski J., Hassan F.M., Mossa H.A., Najim A.A. (2022). Incorporation of digital elevation model, normalized difference vegetation index, and Landsat-8 data for land use land cover mapping. Photogramm. Eng. Remote Sens..

[6] Lakshmi Priya G., Chandra Mouli P., Domnic S., Chemmalar Selvi G., Cho B.K. (2024). Hyperspectral image classification using Walsh Hadamard transform-based key band selection and deep convolutional neural networks. Int. J. Remote Sens..

[7] Byerlay R.A., Nambiar M.K., Nazem A., Nahian M.R., Biglarbegian M., Aliabadi A.A. (2020). Measurement of land surface temperature from oblique angle airborne thermal camera observations. Int. J. Remote Sens..

[8] Alpers W., Kong W., Zeng K., Chan P.W. (2024). On the physical mechanism causing strongly enhanced radar backscatter in C-Band SAR images of convective rain over the ocean. Int. J. Remote Sens..

[9] Zhu X., Cai F., Tian J., Williams T. (2018). Spatiotemporal Fusion of Multisource Remote Sensing Data: Literature Survey, Taxonomy, Principles, Applications, and Future Directions. Remote Sens..

[10] Zhukov B., Oertel D., Lanzl F., Reinhackel G. (1999). Unmixing-based multisensor multiresolution image fusion. IEEE Trans. Geosci. Remote Sens..

[11] Wu M., Wu C., Huang W., Niu Z., Wang C., Li W., Hao P. (2016). An improved high spatial and temporal data fusion approach for combining Landsat and MODIS data to generate daily synthetic Landsat imagery. Inf. Fusion.

[12] Gao F., Masek J., Schwaller M., Hall F. (2006). On the blending of the Landsat and MODIS surface reflectance: Predicting daily Landsat surface reflectance. IEEE Trans. Geosci. Remote Sens..

[13] Zhu X., Chen J., Gao F., Chen X., Masek J.G. (2010). An enhanced spatial and temporal adaptive reflectance fusion model for complex heterogeneous regions. Remote Sens. Environ..

[14] Hilker T., Wulder M.A., Coops N.C., Linke J., McDermid G., Masek J.G., Gao F., White J.C. (2009). A new data fusion model for high spatial- and temporal-resolution mapping of forest disturbance based on Landsat and MODIS. Remote Sens. Environ..

[15] Li A., Bo Y., Zhu Y., Guo P., Bi J., He Y. (2013). Blending multi-resolution satellite sea surface temperature (SST) products using Bayesian maximum entropy method. Remote Sens. Environ..

[16] Huang B., Song H. (2012). Spatiotemporal Reflectance Fusion via Sparse Representation. IEEE Trans. Geosci. Remote Sens..

[17] Zhu X., Helmer E.H., Gao F., Liu D., Chen J., Lefsky M.A. (2016). A flexible spatiotemporal method for fusing satellite images with different resolutions. Remote Sens. Environ..

[18] Moosavi V., Talebi A., Mokhtari M.H., Shamsi S.R.F., Niazi Y. (2015). A wavelet-artificial intelligence fusion approach (WAIFA) for blending Landsat and MODIS surface temperature. Remote Sens. Environ..

[19] Liu X., Deng C., Wang S., Huang G.B., Zhao B., Lauren P. (2016). Fast and Accurate Spatiotemporal Fusion Based Upon Extreme Learning Machine. IEEE Geosci. Remote Sensing Lett..

[20] Fung C.H., Wong M.S., Chan P.W. (2019). Spatio-Temporal Data Fusion for Satellite Images Using Hopfield Neural Network. Remote Sens..

[21] Krizhevsky A., Sutskever I., Hinton G.E. (2017). ImageNet classification with deep convolutional neural networks. Commun. ACM.

[22] Goodfellow I., Pouget-Abadie J., Mirza M., Xu B., Warde-Farley D., Ozair S., Courville A., Bengio Y., Ghahramani Z., Welling M., Cortes C., Lawrence N., Weinberger K. (2014). Generative Adversarial Nets. Proceedings of the Advances in Neural Information Processing Systems.

[23] Vaswani A., Shazeer N., Parmar N., Uszkoreit J., Jones L., Gomez A.N., Kaiser L.U., Polosukhin I., Guyon I., Luxburg U.V., Bengio S., Wallach H., Fergus R., Vishwanathan S., Garnett R. (2017). Attention is All you Need. Proceedings of the Advances in Neural Information Processing Systems.

[24] Ho J., Jain A., Abbeel P. (2020). Denoising Diffusion Probabilistic Models. Advances in Neural Information Processing Systems.

[25] Belgiu M., Stein A. (2019). Spatiotemporal Image Fusion in Remote Sensing. Remote Sens..

[26] Tan Z., Yue P., Di L., Tang J. (2018). Deriving High Spatiotemporal Remote Sensing Images Using Deep Convolutional Network. Remote Sens..

[27] Tan Z., Gao M., Li X., Jiang L. (2021). A Flexible Reference-Insensitive Spatiotemporal Fusion Model for Remote Sensing Images Using Conditional Generative Adversarial Network. IEEE Trans. Geosci. Remote Sens..

[28] Li W., Cao D., Peng Y., Yang C. (2021). MSNet: A Multi-Stream Fusion Network for Remote Sensing Spatiotemporal Fusion Based on Transformer and Convolution. Remote Sens..

[29] Huang H., He W., Zhang H., Xia Y., Zhang L. (2024). STFDiff: Remote sensing image spatiotemporal fusion with diffusion models. Inf. Fusion.

[30] LeCun Y., Boser B., Denker J.S., Henderson D., Howard R.E., Hubbard W., Jackel L.D. (1989). Backpropagation Applied to Handwritten Zip Code Recognition. Neural Comput..

[31] He K., Zhang X., Ren S., Sun J. Deep Residual Learning for Image Recognition. Proceedings of the 2016 IEEE Conference on Computer Vision and Pattern Recognition (CVPR).

[32] Mnih V., Heess N., Graves A., Kavukcuoglu K., Ghahramani Z., Welling M., Cortes C., Lawrence N., Weinberger K. (2014). Recurrent Models of Visual Attention. Proceedings of the Advances in Neural Information Processing Systems.

[33] Song H., Liu Q., Wang G., Hang R., Huang B. (2018). Spatiotemporal Satellite Image Fusion Using Deep Convolutional Neural Networks. IEEE J. Sel. Top. Appl. Earth Obs. Remote Sens..

[34] Li W., Yang C., Peng Y., Zhang X. (2021). A Multi-Cooperative Deep Convolutional Neural Network for Spatiotemporal Satellite Image Fusion. IEEE J. Sel. Top. Appl. Earth Obs. Remote Sens..

[35] Zheng Y., Song H., Sun L., Wu Z., Jeon B. (2019). Spatiotemporal Fusion of Satellite Images via Very Deep Convolutional Networks. Remote Sens..

[36] Peng M., Zhang L., Sun X., Cen Y., Zhao X. (2022). A Synchronous Long Time-Series Completion Method Using 3-D Fully Convolutional Neural Networks. IEEE Geosci. Remote Sens. Lett..

[37] Shao Z., Cai J., Fu P., Hu L., Liu T. (2019). Deep learning-based fusion of Landsat-8 and Sentinel-2 images for a harmonized surface reflectance product. Remote Sens. Environ..

[38] Qin P., Huang H., Tang H., Wang J., Liu C. (2022). MUSTFN: A spatiotemporal fusion method for multi-scale and multi-sensor remote sensing images based on a convolutional neural network. Int. J. Appl. Earth Obs. Geoinf..

[39] Liu X., Deng C., Chanussot J., Hong D., Zhao B. (2019). *StfNet*: A Two-Stream Convolutional Neural Network for Spatiotemporal Image Fusion. IEEE Trans. Geosci. Remote Sens..

[40] Wang X., Shao Z., Huang X., Li D. (2022). Spatiotemporal Temperature Fusion Based on a Deep Convolutional Network. Photogramm Eng Remote Sens..

[41] Jia D., Song C., Cheng C., Shen S., Ning L., Hui C. (2020). A Novel Deep Learning-Based Spatiotemporal Fusion Method for Combining Satellite Images with Different Resolutions Using a Two-Stream Convolutional Neural Network. Remote Sens..

[42] You M., Meng X., Liu Q., Shao F., Fu R. (2024). CIG-STF: Change Information Guided Spatiotemporal Fusion for Remote Sensing Images. IEEE Trans. Geosci. Remote Sens..

[43] Li Y., Li J., He L., Chen J., Plaza A. (2020). A new sensor bias-driven spatio-temporal fusion model based on convolutional neural networks. Sci. China Inf. Sci..

[44] Zhang Y., Liu J., Liang S., Li M. (2022). A New Spatial–Temporal Depthwise Separable Convolutional Fusion Network for Generating Landsat 8-Day Surface Reflectance Time Series over Forest Regions. Remote Sens..

[45] Tan Z., Di L., Zhang M., Guo L., Gao M. (2019). An Enhanced Deep Convolutional Model for Spatiotemporal Image Fusion. Remote Sens..

[46] Li W., Wu F., Cao D. (2022). Dual-Branch Remote Sensing Spatiotemporal Fusion Network Based on Selection Kernel Mechanism. Remote Sens..

[47] Li Y., Liu C., Yan L., Li J., Plaza A., Li B. A New Spatio-Temporal Fusion Method for Remotely Sensed Data Based on Convolutional Neural Networks. Proceedings of the IGARSS 2019—2019 IEEE International Geoscience and Remote Sensing Symposium.

[48] Hoque M.R.U., Wu J., Kwan C., Koperski K., Li J. (2022). ArithFusion: An Arithmetic Deep Model for Temporal Remote Sensing Image Fusion. Remote Sens..

[49] Li W., Zhang X., Peng Y., Dong M. (2020). DMNet: A Network Architecture Using Dilated Convolution and Multiscale Mechanisms for Spatiotemporal Fusion of Remote Sensing Images. IEEE Sensors J..

[50] Jia D., Cheng C., Shen S., Ning L. (2022). Multitask Deep Learning Framework for Spatiotemporal Fusion of NDVI. IEEE Trans. Geosci. Remote Sens..

[51] Peng M., Zhang L., Sun X., Cen Y., Zhao X. (2020). A Fast Three-Dimensional Convolutional Neural Network-Based Spatiotemporal Fusion Method (STF3DCNN) Using a Spatial-Temporal-Spectral Dataset. Remote Sens..

[52] Xiong S., Du S., Zhang X., Ouyang S., Cui W. (2022). Fusing Landsat-7, Landsat-8 and Sentinel-2 surface reflectance to generate dense time series images with 10m spatial resolution. Int. J. Remote. Sens..

[53] Wang X., Wang X. (2020). Spatiotemporal Fusion of Remote Sensing Image Based on Deep Learning. J. Sens..

[54] Fang S., Meng S., Zhang J., Cao Y. (2022). Two-stream spatiotemporal image fusion network based on difference transformation. J. Appl. Remote Sens..

[55] Jia D., Cheng C., Song C., Shen S., Ning L., Zhang T. (2021). A Hybrid Deep Learning-Based Spatiotemporal Fusion Method for Combining Satellite Images with Different Resolutions. Remote Sens..

[56] Cai J., Huang B., Fung T. (2022). Progressive spatiotemporal image fusion with deep neural networks. Int. J. Appl. Earth Obs. Geoinf..

[57] Htitiou A., Boudhar A., Benabdelouahab T. (2021). Deep Learning-Based Spatiotemporal Fusion Approach for Producing High-Resolution NDVI Time-Series Datasets. Can. J. Remote. Sens..

[58] Wei J., Yang H., Tang W., Li Q. (2022). Spatiotemporal-Spectral Fusion for Gaofen-1 Satellite Images. IEEE Geosci. Remote Sensing Lett..

[59] Wei J., Tang W., He C. (2021). Enblending Mosaicked Remote Sensing Images with Spatiotemporal Fusion of Convolutional Neural Networks. IEEE J. Sel. Top. Appl. Earth Obs. Remote Sens..

[60] Erdem F., Avdan U. (2023). STFRDN: A residual dense network for remote sensing image spatiotemporal fusion. Int. J. Remote Sens..

[61] Wang J., Chen F., Zhang M., Yu B. (2021). ACFNet: A Feature Fusion Network for Glacial Lake Extraction Based on Optical and Synthetic Aperture Radar Images. Remote Sens..

[62] Xiao J., Aggarwal A.K., Rage U.K., Katiyar V., Avtar R. (2023). Deep Learning-Based Spatiotemporal Fusion of Unmanned Aerial Vehicle and Satellite Reflectance Images for Crop Monitoring. IEEE Access.

[63] Bai Y., Wu W., Yang Z., Yu J., Zhao B., Liu X., Yang H., Mas E., Koshimura S. (2021). Enhancement of Detecting Permanent Water and Temporary Water in Flood Disasters by Fusing Sentinel-1 and Sentinel-2 Imagery Using Deep Learning Algorithms: Demonstration of Sen1Floods11 Benchmark Datasets. Remote Sens..

[64] Zeng Y., Gao B., Liu P., Zhao X. (2024). Spatiotemporal Fusion for Nighttime Light Remote Sensing Images With Multivariate Activation Function. IEEE Geosci. Remote Sens. Lett..

[65] Yin Z., Wu P., Foody G.M., Wu Y., Liu Z., Du Y., Ling F. (2021). Spatiotemporal Fusion of Land Surface Temperature Based on a Convolutional Neural Network. IEEE Trans. Geosci. Remote Sens..

[66] Fu R., Hu H., Wu N., Liu Z., Jin W. (2024). Spatiotemporal fusion convolutional neural network: Tropical cyclone intensity estimation from multisource remote sensing images. J. Appl. Remote Sens..

[67] Chen Y., Shi K., Ge Y., Zhou Y. (2022). Spatiotemporal Remote Sensing Image Fusion Using Multiscale Two-Stream Convolutional Neural Networks. IEEE Trans. Geosci. Remote Sens..

[68] Li W., Zhang X., Peng Y., Dong M. (2021). Spatiotemporal Fusion of Remote Sensing Images using a Convolutional Neural Network with Attention and Multiscale Mechanisms. Int. J. Remote. Sens..

[69] Wu J., Lin L., Li T., Cheng Q., Zhang C., Shen H. (2022). Fusing Landsat 8 and Sentinel-2 data for 10-m dense time-series imagery using a degradation-term constrained deep network. Int. J. Appl. Earth Obs. Geoinf..

[70] Ao Z., Sun Y., Xin Q. (2021). Constructing 10-m NDVI Time Series From Landsat 8 and Sentinel 2 Images Using Convolutional Neural Networks. IEEE Geosci. Remote Sens. Lett..

[71] Wang S., Cui D., Wang L., Peng J. (2023). Applying deep-learning enhanced fusion methods for improved NDVI reconstruction and long-term vegetation cover study: A case of the Danjiang River Basin. Ecol. Indic..

[72] Li W., Yang C., Peng Y., Du J. (2022). A Pseudo-Siamese Deep Convolutional Neural Network for Spatiotemporal Satellite Image Fusion. IEEE J. Sel. Top. Appl. Earth Obs. Remote Sens..

[73] Lin L., Shen Y., Wu J., Nan F. (2023). CAFE: A Cross-Attention Based Adaptive Weighting Fusion Network for MODIS and Landsat Spatiotemporal Fusion. IEEE Geosci. Remote Sens. Lett..

[74] Sun H., Xiao W. (2022). Similarity Weight Learning: A New Spatial and Temporal Satellite Image Fusion Framework. IEEE Trans. Geosci. Remote Sens..

[75] Cai Z., Hu Q., Zhang X., Yang J., Wei H., Wang J., Zeng Y., Yin G., Li W., You L. (2023). Improving agricultural field parcel delineation with a dual branch spatiotemporal fusion network by integrating multimodal satellite data. ISPRS J. Photogramm. Remote. Sens..

[76] Cheng F., Fu Z., Tang B., Huang L., Huang K., Ji X. (2022). STF-EGFA: A Remote Sensing Spatiotemporal Fusion Network with Edge-Guided Feature Attention. Remote Sens..

[77] Ran Q., Wang Q., Zheng K., Li J. (2024). Multiscale Attention Spatiotemporal Fusion Model Based on Pyramidal Network Constraints. IEEE Geosci. Remote Sens. Lett..

[78] Lei D., Huang Z., Zhang L., Li W. (2022). SCRNet: An efficient spatial channel attention residual network for spatiotemporal fusion. J. Appl. Remote Sens..

[79] Zhang X., Li S., Tan Z., Li X. (2024). Enhanced wavelet based spatiotemporal fusion networks using cross-paired remote sensing images. ISPRS J. Photogramm. Remote Sens..

[80] Cao H., Luo X., Peng Y., Xie T. (2022). MANet: A Network Architecture for Remote Sensing Spatiotemporal Fusion Based on Multiscale and Attention Mechanisms. Remote Sens..

[81] Cui D., Wang S., Zhao C., Zhang H. (2024). A Novel Remote Sensing Spatiotemporal Data Fusion Framework Based on the Combination of Deep-Learning Downscaling and Traditional Fusion Algorithm. IEEE J. Sel. Top. Appl. Earth Obs. Remote Sens..

[82] Yang Z., Diao C., Li B. (2021). A Robust Hybrid Deep Learning Model for Spatiotemporal Image Fusion. Remote Sens..

[83] Zhan W., Luo F., Luo H., Li J., Wu Y., Yin Z., Wu Y., Wu P. (2024). Time-Series-Based Spatiotemporal Fusion Network for Improving Crop Type Mapping. Remote Sens..

[84] Wei J., Chen L., Chen Z., Huang Y. (2023). An Experimental Study of the Accuracy and Change Detection Potential of Blending Time Series Remote Sensing Images with Spatiotemporal Fusion. Remote Sens..

[85] Zheng X., Feng R., Fan J., Han W., Yu S., Chen J. (2023). MSISR-STF: Spatiotemporal Fusion via Multilevel Single-Image Super-Resolution. Remote Sens..

[86] Ronneberger O., Fischer P., Brox T., Navab N., Hornegger J., Wells W.M., Frangi A.F. (2015). U-Net: Convolutional Networks for Biomedical Image Segmentation. Medical Image Computing and Computer-Assisted Intervention—MICCAI 2015.

[87] Zhou J., He Z., Song Y.N., Wang H., Yang X., Lian W., Dai H.N. (2020). Precious Metal Price Prediction Based on Deep Regularization Self-Attention Regression. IEEE Access.

[88] Talbi F., Chikr Elmezouar M., Boutellaa E., Alim F. (2023). Vector-Quantized Variational AutoEncoder for pansharpening. Int. J. Remote Sens..

[89] Chen J., Wang L., Feng R., Liu P., Han W., Chen X. (2020). CycleGAN-STF: Spatiotemporal Fusion via CycleGAN-Based Image Generation. IEEE Trans. Geosci. Remote Sens..

[90] Tan Z., Gao M., Yuan J., Jiang L., Duan H. (2022). A Robust Model for MODIS and Landsat Image Fusion Considering Input Noise. IEEE Trans. Geosci. Remote Sens..

[91] Ma Y., Wei J., Tang W., Tang R. (2021). Explicit and stepwise models for spatiotemporal fusion of remote sensing images with deep neural networks. Int. J. Appl. Earth Obs. Geoinf..

[92] Wang Y., Gu L., Li X., Gao F., Jiang T., Ren R. (2022). An Improved Spatiotemporal Fusion Algorithm for Monitoring Daily Snow Cover Changes with High Spatial Resolution. IEEE Trans. Geosci. Remote Sens..

[93] Liu H., Yang G., Deng F., Qian Y., Fan Y. (2023). MCBAM-GAN: The Gan Spatiotemporal Fusion Model Based on Multiscale and CBAM for Remote Sensing Images. Remote Sens..

[94] Zhang H., Song Y., Han C., Zhang L. (2021). Remote Sensing Image Spatiotemporal Fusion Using a Generative Adversarial Network. IEEE Trans. Geosci. Remote Sens..

[95] Pan X., Deng M., Ao Z., Xin Q. (2023). An Adaptive Multiscale Generative Adversarial Network for the Spatiotemporal Fusion of Landsat and MODIS Data. Remote Sens..

[96] Fang S., Guo Q., Cao Y., Zhang J. A Two-Layers Super-Resolution Based Generation Adversarial Spatiotemporal Fusion Model. Proceedings of the IGARSS 2022—2022 IEEE International Geoscience and Remote Sensing Symposium.

[97] Sun W., Li J., Jiang M., Yuan Q. (2023). Supervised and self-supervised learning-based cascade spatiotemporal fusion framework and its application. ISPRS J. Photogramm. Remote. Sens..

[98] Jiang M., Shen H., Li J. (2022). Deep-Learning-Based Spatio-Temporal-Spectral Integrated Fusion of Heterogeneous Remote Sensing Images. IEEE Trans. Geosci. Remote Sens..

[99] Wu Y., Feng S., Huang M. (2023). An enhanced spatiotemporal fusion model with degraded fine-resolution images via relativistic generative adversarial networks. Geocarto Int..

[100] Liu Q., Meng X., Shao F., Li S. (2022). PSTAF-GAN: Progressive Spatio-Temporal Attention Fusion Method Based on Generative Adversarial Network. IEEE Trans. Geosci. Remote Sens..

[101] Wu Y., Li Y., Huang M., Feng S. (2023). Multiresolution generative adversarial networks with bidirectional adaptive-stage progressive guided fusion for remote sensing image. Int. J. Digit. Earth.

[102] Ma Y., Wei J., Huang X. (2022). Balancing Colors of Nonoverlapping Mosaicking Images with Generative Adversarial Networks. IEEE Geosci. Remote Sens. Lett..

[103] Liu S., Liu J., Tan X., Chen X., Chen J. (2024). A Hybrid Spatiotemporal Fusion Method for High Spatial Resolution Imagery: Fusion of Gaofen-1 and Sentinel-2 over Agricultural Landscapes. J. Remote Sens..

[104] Shang C., Li X., Yin Z., Li X., Wang L., Zhang Y., Du Y., Ling F. (2022). Spatiotemporal Reflectance Fusion Using a Generative Adversarial Network. IEEE Trans. Geosci. Remote Sens..

[105] Sun W., Ren K., Meng X., Yang G., Liu Q., Zhu L., Peng J., Li J. (2024). Generating high-resolution hyperspectral time series datasets based on unsupervised spatial-temporal-spectral fusion network incorporating a deep prior. Inf. Fusion.

[106] Song B., Liu P., Li J., Wang L., Zhang L., He G., Chen L., Liu J. (2022). MLFF-GAN: A Multilevel Feature Fusion With GAN for Spatiotemporal Remote Sensing Images. IEEE Trans. Geosci. Remote Sens..

[107] Weng C., Zhan Y., Gu X., Yang J., Liu Y., Guo H., Lian Z., Zhang S., Wang Z., Zhao X. (2024). The Spatially Seamless Spatiotemporal Fusion Model Based on Generative Adversarial Networks. IEEE J. Sel. Top. Appl. Earth Obs. Remote Sens..

[108] Song Y., Zhang H., Huang H., Zhang L. (2022). Remote Sensing Image Spatiotemporal Fusion via a Generative Adversarial Network With One Prior Image Pair. IEEE Trans. Geosci. Remote Sens..

[109] Dosovitskiy A., Beyer L., Kolesnikov A., Weissenborn D., Zhai X., Unterthiner T., Dehghani M., Minderer M., Heigold G., Gelly S. (2021). An Image is Worth 16x16 Words: Transformers for Image Recognition at Scale. arXiv.

[110] Gulrajani I., Ahmed F., Arjovsky M., Dumoulin V., Courville A.C. Improved Training of Wasserstein GANs. Proceedings of the Advances in Neural Information Processing Systems.

[111] Yang G., Qian Y., Liu H., Tang B., Qi R., Lu Y., Geng J. (2022). MSFusion: Multistage for Remote Sensing Image Spatiotemporal Fusion Based on Texture Transformer and Convolutional Neural Network. IEEE J. Sel. Top. Appl. Earth Obs. Remote Sens..

[112] Chen G., Jiao P., Hu Q., Xiao L., Ye Z. (2022). SwinSTFM: Remote Sensing Spatiotemporal Fusion Using Swin Transformer. IEEE Trans. Geosci. Remote Sens..

[113] Wang Z., Fang S., Zhang J. (2023). Spatiotemporal Fusion Model of Remote Sensing Images Combining Single-Band and Multi-Band Prediction. Remote Sens..

[114] Li W., Cao D., Xiang M. (2022). Enhanced Multi-Stream Remote Sensing Spatiotemporal Fusion Network Based on Transformer and Dilated Convolution. Remote Sens..

[115] Benzenati T., Kallel A., Kessentini Y. (2024). STF-Trans: A two-stream spatiotemporal fusion transformer for very high resolution satellites images. Neurocomputing.

[116] Qian Z., Yue L., Xie X., Yuan Q., Shen H. (2024). A Dual-Perspective Spatiotemporal Fusion Model for Remote Sensing Images by Discriminative Learning of the Spatial and Temporal Mapping. IEEE J. Sel. Top. Appl. Earth Obs. Remote Sens..

[117] Liu H., Qian Y., Yang G., Jiang H. (2022). Super-Resolution Reconstruction Model of Spatiotemporal Fusion Remote Sensing Image Based on Double Branch Texture Transformers and Feedback Mechanism. Electronics.

[118] Ramesh A., Pavlov M., Goh G., Gray S., Voss C., Radford A., Chen M., Sutskever I. Zero-Shot Text-to-Image Generation. Proceedings of the International Conference on Machine Learning.

[119] Moser B.B., Shanbhag A.S., Raue F., Frolov S., Palacio S., Dengel A. (2024). Diffusion Models, Image Super-Resolution and Everything: A Survey. IEEE Trans. Neural Netw. Learn. Syst..

[120] Kulikov V., Yadin S., Kleiner M., Michaeli T. Sinddm: A single image denoising diffusion model. Proceedings of the International Conference on Machine Learning, PMLR.

[121] Li X., Ren Y., Jin X., Lan C., Wang X., Zeng W., Wang X., Chen Z. (2023). Diffusion Models for Image Restoration and Enhancement—A Comprehensive Survey. arXiv.

[122] Ma Y., Wang Q., Wei J. (2024). Spatiotemporal Fusion via Conditional Diffusion Model. IEEE Geosci. Remote Sens. Lett..

[123] Wei J., Gan L., Tang W., Li M., Song Y. (2024). Diffusion models for spatio-temporal-spectral fusion of homogeneous Gaofen-1 satellite platforms. Int. J. Appl. Earth Obs. Geoinf..

[124] Han W., Li J., Wang S., Zhang X., Dong Y., Fan R., Zhang X., Wang L. (2022). Geological Remote Sensing Interpretation Using Deep Learning Feature and an Adaptive Multisource Data Fusion Network. IEEE Trans. Geosci. Remote Sens..

[125] Elizar E., Zulkifley M.A., Muharar R., Zaman M.H.M., Mustaza S.M. (2022). A Review on Multiscale-Deep-Learning Applications. Sensors.

[126] Swain R., Paul A., Behera M.D. (2023). Spatio-temporal fusion methods for spectral remote sensing: A comprehensive technical review and comparative analysis. Trop. Ecol..

[127] Xue J., Leung Y., Fung T. (2017). A Bayesian Data Fusion Approach to Spatio-Temporal Fusion of Remotely Sensed Images. Remote Sens..

[128] Zhou J., Chen J., Chen X., Zhu X., Qiu Y., Song H., Rao Y., Zhang C., Cao X., Cui X. (2021). Sensitivity of six typical spatiotemporal fusion methods to different influential factors: A comparative study for a normalized difference vegetation index time series reconstruction. Remote Sens. Environ..

[129] Li J., Hong D., Gao L., Yao J., Zheng K., Zhang B., Chanussot J. (2022). Deep learning in multimodal remote sensing data fusion: A comprehensive review. Int. J. Appl. Earth Obs. Geoinf..

[130] Zhu X., Zhan W., Zhou J., Chen X., Liang Z., Xu S., Chen J. (2022). A novel framework to assess all-round performances of spatiotemporal fusion models. Remote Sens. Environ..

[131] Guo D., Shi W., Qian F., Wang S., Cai C. (2022). Monitoring the spatiotemporal change of Dongting Lake wetland by integrating Landsat and MODIS images, from 2001 to 2020. Ecol. Inform..

[132] Emelyanova I.V., McVicar T.R., Van Niel T.G., Li L.T., Van Dijk A.I. (2013). Assessing the accuracy of blending Landsat—MODIS surface reflectances in two landscapes with contrasting spatial and temporal dynamics: A framework for algorithm selection. Remote Sens. Environ..

[133] Li J., Li Y., He L., Chen J., Plaza A. (2020). Spatio-temporal fusion for remote sensing data: An overview and new benchmark. Sci. China Inf. Sci..

[134] Guo D., Shi W. (2023). Object-Level Hybrid Spatiotemporal Fusion: Reaching a Better Tradeoff Among Spectral Accuracy, Spatial Accuracy, and Efficiency. IEEE J. Sel. Top. Appl. Earth Obs. Remote Sens..

[135] Tasar O., Tarabalka Y., Alliez P. (2019). Incremental Learning for Semantic Segmentation of Large-Scale Remote Sensing Data. IEEE J. Sel. Top. Appl. Earth Obs. Remote Sens..

[136] Yin M., Chen Z., Zhang C. (2023). A CNN-Transformer Network Combining CBAM for Change Detection in High-Resolution Remote Sensing Images. Remote Sens..

